# A free-boundary model of a motile cell explains turning behavior

**DOI:** 10.1371/journal.pcbi.1005862

**Published:** 2017-11-14

**Authors:** Masoud Nickaeen, Igor L. Novak, Stephanie Pulford, Aaron Rumack, Jamie Brandon, Boris M. Slepchenko, Alex Mogilner

**Affiliations:** 1 Richard D. Berlin Center for Cell Analysis and Modeling, Department of Cell Biology, University of Connecticut Health Center, Farmington, CT, United States of America; 2 Center for Engineering Learning & Teaching, University of Washington, Seattle, WA, United States of America; 3 Department of Computer Science, Cornell University, Ithaca, NY, United States of America; 4 Department of Mathematics, Adrian College, Adrian, MI, United States of America; 5 Courant Institute and Department of Biology, New York University, New York, NY, United States of America; National Institutes of Health, UNITED STATES

## Abstract

To understand shapes and movements of cells undergoing lamellipodial motility, we systematically explore minimal free-boundary models of actin-myosin contractility consisting of the force-balance and myosin transport equations. The models account for isotropic contraction proportional to myosin density, viscous stresses in the actin network, and constant-strength viscous-like adhesion. The contraction generates a spatially graded centripetal actin flow, which in turn reinforces the contraction via myosin redistribution and causes retraction of the lamellipodial boundary. Actin protrusion at the boundary counters the retraction, and the balance of the protrusion and retraction shapes the lamellipodium. The model analysis shows that initiation of motility critically depends on three dimensionless parameter combinations, which represent myosin-dependent contractility, a characteristic viscosity-adhesion length, and a rate of actin protrusion. When the contractility is sufficiently strong, cells break symmetry and move steadily along either straight or circular trajectories, and the motile behavior is sensitive to conditions at the cell boundary. Scanning of a model parameter space shows that the contractile mechanism of motility supports robust cell turning in conditions where short viscosity-adhesion lengths and fast protrusion cause an accumulation of myosin in a small region at the cell rear, destabilizing the axial symmetry of a moving cell.

## Author summary

To understand shapes and movements of simple motile cells, we systematically explore minimal models describing a cell as a two-dimensional actin-myosin gel with a free boundary. The models account for actin-myosin contraction balanced by viscous stresses in the actin gel and uniform adhesion. The myosin contraction causes the lamellipodial boundary to retract. Actin protrusion at the boundary counters the retraction, and the balance of protrusion and retraction shapes the cell. The models reproduce a variety of motile shapes observed experimentally. The analysis shows that the mechanical state of a cell depends on a small number of parameters. We find that when the contractility is sufficiently strong, cells break symmetry and move steadily along either straight or circular trajectory. Scanning model parameters shows that the contractile mechanism of motility supports robust cell turning behavior in conditions where deformable actin gel and fast protrusion destabilize the axial symmetry of a moving cell.

## Introduction

Cell motility is a fundamental biological phenomenon that underlies many physiological processes in health and disease, including wound healing, embryogenesis, immune response, and metastatic spread of cancer cells [1], to name a few. Understanding the full complexity of cell motility, exacerbated by complex biochemical regulation, poses enormous challenges. One of them is multiple, sometimes redundant, sometimes complementary or even competing, mechanisms of motility [2]. Many researchers hold the view, which we share, that the way to face this challenge is to study all these mechanisms thoroughly, and then proceed with a more holistic approach.

One of the best studied types of motility is the lamellipodial motility on flat, hard and adhesive surfaces [3], in which broad and flat motile appendages–lamellipodia–spread around the cell body. Biochemical regulation plays an important role in the lamellipodial dynamics, but minimal mechanisms of the lamellipodial motility, such as growth and spreading of a flat actin network wrapped in plasma membrane and myosin-powered contraction of this network, are mechanical in nature [3]. While many cell types exhibit the lamellipodial motility, one model system, the fish epithelial keratocyte cell, contributed very prominently to the understanding of lamellipodial mechanics, due to its large lamellipodium, streamlined for rapid and steady locomotion [4, 5].

There are at least three distinct mechanical states of this system. The cells can be in a stationary symmetric state, with a ring-like lamellipodium around the cell body [6]. Spontaneously, even if slowly, the cells self-polarize, so that the lamellipodium retracts at the prospective rear and takes on a fan-like shape, upon which the cell starts crawling with a constant speed and steady shape [6, 7]. Often, cell’s trajectory changes from straight to circular–the cells start turning [8].

Mechanics of keratocyte movements has been studied extensively [4, 5, 7, 9]. Two principal mechanisms enable the keratocyte motility. First, polymerization of the polarized actin network at the front pushes forward the membrane at the leading edge, stretching the membrane and creating membrane tension at the sides; the membrane then snaps at the rear and pulls forward the depolymerizing actin network [10]. Second, contractile forces generated by myosin, lagging behind in a moving cell, hold the cell sides and retract the rear, allowing the front to protrude [5]. This and stick-slip dynamics of adhesions were recently shown to generate the cell self-polarization [7].

One of the fundamental questions of cell motility concerns dynamics of the cell shape: how do the actin-myosin mechanics in the cell bulk interact with actin growth and membrane mechanics at the boundary to shape, stabilize and propel the cell? This question requires mathematical insight, and in the last two decades, keratocyte mechanics were extensively modeled mathematically. The mathematical problem arising in these models is generally challenging, given that the motile cell is a free-boundary object, in which deformations of the cell shape depend on, and in turn affect, the actin-myosin movements and forces inside the cell. The history of the free-boundary cell modeling was recently reviewed in [11].

To reduce the mathematical complexity of the problem, one can ignore the mechanics in the lateral cross section and consider a simplified one-dimensional (1D) model, essentially representing the cell as a 1D strip of an actin-myosin gel. Mathematical models of this kind [12–15] provided valuable insight into conditions for symmetry breaking, motility initiation, and stabilization of the anterior-posterior length of the moving cell. Modeling the front-to-rear cell mechanics is not the only 1D approach: one can also disregard the bulk of the actin-myosin network and hypothesize that the essential dynamics is concentrated at the very edge of the cell; this allows one to approximate the cell shape by a 1D dynamic contour, which protrudes or retracts locally according to some set of rules. A number of such models [16–19] revealed that a small set of the boundary deformation rules can generate an unexpected variety of dynamic cell shapes mimicking a number of observed motile cell types. The first such model was a Graded Radial Extension mathematical model [20], which integrated experimental data and posited that actin polymerization at the lamellipodial boundary results in protrusion of the cell front and sides in the direction locally normal to the boundary, with spatially graded rate maximal at the center of the leading edge and decreasing towards the sides.

A more accurate mathematical rendering of the lamellipodia is achieved via a full 2D free-boundary model. Its general concept, first introduced in [21], is as follows. Actin-myosin contraction in the bulk of the 2D lamellipodium generates a centripetal actin flow that redistributes myosin powering the contraction; this feedback results in a spatially graded flow that tends to retract the lamellipodial boundary. Actin growth at the boundary results in protrusion countering the retraction, and the balance of protrusion and retraction shapes the lamellipodium, feeding back to the actin-myosin contraction in the bulk of the lamellipodium. The question is: what kind of cell shapes and movements does this model predict?

To address this question, 2D models of actin-myosin mechanics were employed to reproduce steady-state shapes and speed, as well as self-polarization, of a motile cell [5, 7, 22], but a number of important issues have not been adequately explored, including turning behavior and dependence of the motile behavior on the model parameters and boundary conditions. In this paper, we resolve these issues by simulating numerically a minimal free-boundary model described in the next section. We find that 1) cells are stationary when contractility is weak, 2) when contractility is strong, cells break symmetry and move steadily along either straight or circular trajectory, 3) cells exhibit turning behavior when fast protrusion destabilizes the axial symmetry of a planar myosin distribution or cell shape, and 4) motile behavior of a cell is sensitive to conditions of force balance at the cell boundary.

## Models and methods

Mechanics plays a dominant role in keratocyte motility, while the role of biochemical regulation is less clear, and is probably of less importance [3]; thus we focus on mechanical formulation. Moreover, at least for the self-polarization phenomenon, the contractile mechanism of motility is dominant compared to the graded actin polymerization [7], and so we concentrate on the myosin generated forces and movements, and for simplicity assume that the actin growth is uniform around the cell boundary.

The model consists of force-balance and myosin transport equations,

$$\begin{array}{l} \eta \Delta_{X}U+\sigma \nabla_{X}M-\xi U=0\\ \partial_{T}M=\nabla_{X}\cdot (D_{\mathrm{eff}}\nabla_{X}M-U_{\mathrm{eff}}M) \end{array}$$ (1)

for the velocity of actin flow **U**(**X**,*t*) and myosin concentration *M*(**X**,*t*) defined locally for **X** ∈ *Ω*(*t*), where *Ω*(*t*) is a moving 2D domain representing cell geometry. Note, that all model equations are formulated in the lab frame of references. We discuss the validity of approximating the flat lamellipodium as a 2D domain in the thin-cell limit in the Supplemental Material. The model is similar to an actomyosin dynamic model suggested in [5, 23], and to active gel models of the soft matter physics [24]. In the force balance equation, the first term describes the force due to passive viscous stresses in the deforming actin network, where *η* is the effective actin viscosity. The form of this term corresponds to the viscous shear stress in the Stokes equation of hydrodynamics; we emphasize that the actin polymer mesh is compressible (fluid cytoplasm can be squeezed easily into the dorsal direction in the cell [23]), and so there is no incompressibility condition. In the Supplemental Material, we discuss the conditions under which the effects of hydrostatic pressure and Darcy flow are negligible in the lamellipodium. Because the movement of the cell takes place on the slow time scales, we do not consider viscoelastic effects [23]. Also, as was done in many modeling studies [25], we ignore for simplicity subtle and complex interplay between bulk and shear viscous stresses.

The second term in the force-balance equation describes the divergence of the myosin contractile stress. As in [5, 23, 24], we assume the stress to be isotropic and proportional to the myosin density, with *σ* denoting the force per unit of myosin density. The third term describes the effective viscous drag arising from creeping movement of F-actin relative to a substrate, mediated by dynamic adhesions and characterized by the viscous drag coefficient *ξ*. The linear dependence of this drag on actin velocity is a standard assumption made in cell mechanics models; for some cases, this assumption was verified experimentally.

The second of Eq (1) describes myosin transport. Kinetics of myosin can be interpreted in terms of transitions between two states, a state of free myosin diffusing in the cytoplasm and a state in which myosin is bound to the actin network [23]; clusters of the bound myosin both contract the actin network and move with it. For the transitions occurring on a fast time scale, the overall transport is well approximated by a diffusion-advection equation, and we assume this limit in our model here. Additional discussion of the myosin transport is in the Supplemental Material. For low viscosity and slow diffusion, however, using **U** as an advection velocity and constant diffusion coefficients *D* results in singular solutions, in which *M* and **U** develop Dirac-delta singularities. The effect is reminiscent of the collapsing phenomenon in a 2D version of the Keller-Segel chemotaxis model [26], which is mathematically similar to our mechanical model. The singular solutions are obviously unrealistic, given that myosin molecules have a finite size. The excluded volume effect can be taken into account by introducing effective velocities [27], **U**_eff_ = **U**(1−*M*/*M*_max,*u*_), which approach zero when *M* → *M*_max,*u*_ But in a free-boundary problem, the actual maximum of myosin concentrations may significantly exceed *M*_max,*u*_. This is because in motile solutions, myosin accumulates at the rear of the cell, where it is also swept forward by a moving boundary; mathematically, this effect originates from the Rankine-Hugoniot boundary condition described in the next paragraph. Thus, the effect of molecular crowding on myosin velocity should generally be written as: **U**_eff_ = **U**(1−*M*/*M*_max,*u*_), if *M* < *M*_max,*u*_, and zero otherwise. Because diffusion is also affected by the crowding, the effective diffusivity in Eq (1) is *D*_eff_ = *D*(1−*M*/*M*_max,*d*_), with a value of the cut-off *M*_max,*d*_ that exceeds the actual maximal densities of myosin [28]. Overall, using a tighter myosin cut-off for advection, *M*_max,*u*_ < *M*_max,*d*_, helps avoiding the singularities and numerical instabilities associated with them in a wide range of model parameters. We also explored the possibility that the anti-crowding effects come from an attenuation of the myosin stress when the myosin density is too high, described in detail in the Supplemental Material.

One of our goals is to investigate how boundary conditions, specified in a free boundary problem at a moving cell boundary *∂Ω*(*t*), affect the model behavior. We explore two types of conditions for the force-balance equation. One of them is the zero actin velocity at the boundary, **U**|_*∂Ω*(*t*)_ = 0, which assumes a very narrow band of very strong adhesions near the cell edge that rapidly adjust their positions to the instantaneous location of the boundary. Many experimental observations indicate the presence of such a band. We will term this version of the model a zero-velocity (ZV) model. Alternatively, in the absence of the sticky band, the force balance is reflected by a zero stress condition, introduced earlier in [5, 23]: $n\cdot (\eta \nabla_{X}U+M\hat{I}){|}_{\partial \Omega (t)}=0$, where $\hat{I}$ is the unit tensor and **n** is the outward normal. This assumes that the membrane tension is small relative to the contractile and viscous stresses. We will call this variant of the model a zero-stress (ZS) model. Both versions of the model share a no-flux Rankine-Hugoniot boundary condition for myosin, **n** ⋅(−*D*_eff_ ∇_X_*M* + (**U**_eff_ – **V**_f_)*M*) |_*∂Ω*(*t*)_ = 0, where **V**_f_ is the local boundary velocity.

Kinematics of the boundary is modeled by a superposition of locally normal protrusion powered by actin growth and retraction stemming from the centripetal actin flow: **V**_*f*_ = *V*_p_**n** + **U**|_*∂Ω*(*t*)_. The approximation of the speed of normal protrusion *V*_p_ is somewhat different in the two versions of the model, as described below.

In the ZS model, *V*_p_ is defined uniformly along the boundary but depends on the cell size: *V*_p_ = *V*_0_(*A*_0_/*A*)−*K*(*A*−*A*_0_(*A*_0_/*A*)^*n*^), where *A* = |*Ω*(*t*)|, *A*_0_ = |*Ω*(0)|, and *n* = 2. The first term represents the rate of membrane displacement due to actin growth with a rate constant *V*_0_. The cell-size dependence of this term reflects an effective drop in actin concentration in an expanding cell, but this term alone would still produce an infinite cell expansion for large *V*_0_. Realistically, cell stretching is limited by membrane tension, which is represented in *V*_p_ by −*KA* term; this is consistent with previous experiments and modeling [4, 5, 29]. The term ∝(*A*_0_/*A*)^*n*^ reflects cytoplasmic resistance to contractile forces and thus excludes collapsing of the cell in the model with small *V*_0_. Mathematically, the quadratic nonlinearity in the area dependence appears to be the lowest nonlinearity preventing the cell collapse in the ZS model. Overall, the second term in *V*_p_, combining the effects of membrane tension and cytoplasm resistance to contraction, plays an area-preserving role (with the parameter *K* describing sensitivity of *V*_p_ to changes in *A*). Indeed, if *A*<*A*_0_, the membrane tension decreases, whereas actin polymerization accelerates and the resistance to further contraction rapidly grows. On the other hand, if *A*>*A*_0_, the membrane tension increases rapidly stopping the actin growth.

For the ZV model, where **U**|_*∂Ω*(*t*)_ = 0 and **V**_*f*_ = *V*_p_**n**, there must be a nontrivial variation of *V*_p_ along the boundary, since for a uniform *V*_p_, the cell centroid is always stationary. Based on experimental observations and models showing that myosin can impede protrusion by bundling actin filaments at the boundary [18], we hypothesized that the actin growth rate is a decreasing function of local myosin density. Correspondingly, we use the following expression for *V*_p_ in the ZV model, *V*_p_ = *V*_0_(*A*_0_/*A*)(1+*M*)|_*∂Ω*(*t*)_/M_0_)^−1^−*K*(*A*−*A*_0_), where *M*_0_ is a threshold beyond which myosin inhibits actin growth almost entirely. The expression has essentially the same dependence on cell area as in the ZS model, except that for the ZV model, *n* = 0 proved to be sufficient for preserving the target area. We discuss derivation of the mathematical expression for *V*_p_ from the force balance at the lamellipodial boundary and provide additional explanations in the Supplemental Material.

### Nondimensionalization

To nondimensionalize the model, we use the following set of units. The length unit *L* is defined as a characteristic linear size of the cell with a target area, $L=\sqrt{A_{0}/\pi }$ (e.g., if this cell is a circle, *L* is its radius). We further choose *L*^2^*D*^−1^ and *M*_0_ as the units of time and myosin concentration, respectively. Then the dimensionless variables, differential operator, and current and target cell areas are, respectively, *t* = *TDL*^−2^, **x** = **X***L*^−1^, **u** = **U***LD*^−1^, *m* = *M*/*M*_0_, ∇ ≡ ∇_x_ = *L*∇_X_, |*ω*|=|*Ω*|*L*^−2^ and *a*_0_ = *A*_0_*L*^−2^. Correspondingly, Eqs (1) takes the form,

$$\begin{array}{l} \partial_{t}m=\nabla \cdot (d_{\mathrm{eff}}\nabla m-u_{\mathrm{eff}}m)\\ \alpha \Delta u+\beta \nabla m-u=0 \end{array},$$ (2)

where **u**_eff_ = **u**(1−*m*/*m*_max,*u*_), if *m*<*m*_max,*u*_, and zero otherwise, and *d*_eff_ = 1−*m*/*m*_max,*d*_. Eqs (2) include two dimensionless parameters: the dimensionless viscosity-adhesion length parameter *α* = *ηL*^−2^*ξ*^−1^ and the myosin contractility constant *β* = *σM*_0_(*Dξ*)^−1^. Note that the mechanical effect of localized myosin contraction spreads on the length scale $\sqrt{\eta /\xi }$, so the viscosity-adhesion length parameter *α* is the ratio of the length scale of the mechanical action to the cell size. The first of Eqs (2), a diffusion-advection equation for myosin, is subject to the mass-conserving zero-flux boundary condition, **n** ⋅(−*d*_eff_∇*m*+(**u**_eff_−**v**_f_)*m*)|_*∂ω*(*t*)_ = 0, yielding an additional dimensionless parameter $\mu_{\mathrm{tot}}=\underset{\omega (t)}{\iint }m\cdot d|\omega |$. The dimensionless boundary conditions for the force-balance equation (the second of Eqs (2)) in the ZV and ZS models are **u**|_*∂ω*(*t*)_ = 0 and $n\cdot (\alpha \nabla u+\beta m\hat{I}){|}_{\partial \omega (t)}=0$, respectively.

The dimensionless boundary velocity equation is **v**_*f*_ = *v*_p_**n**+**u**|_*∂ω*(*t*)_. In this equation, the dimensionless rate of membrane displacement caused by the actin polymerization and area preserving factors is *v*_p_ = *v*_0_*a*_0_/*a*−*k*(*a*−*a*_0_(*a*_0_/*a*)^*n*^), where *v*_0_ = *V*_0_*L*/*D* and *a* = |*ω*(*t*)|. For the ZS model, *n* = 2, whereas for the ZV model, *n* = 0 and there is the additional dependence on *m* in the first term: *v*_p_ = *v*_0_*a*_0_/(*a*(1+*m*|_*∂ω*(*t*)_))−*k*(*a*−*a*_0_).

Note that varying parameter *k* = *KL*^3^*D*^−1^ is equivalent to rescaling the actin polymerization constant *v*_0_. Also, because the myosin contractility constant *β* enters Eq (2) in combination with *m*, varying *β* is similar to rescaling *μ*_tot_; in fact, *β* could be formally excluded from the ZS model by employing a different concentration unit, and the same is true for the ZV model defined in a fixed geometry, see section *Cell becomes motile when myosin contractility is higher than critical*. We therefore focus in our study on the role played by three essentially independent model parameters: *α*, *μ*_tot_ and *v*_0_.

### Initial conditions

Steady dynamics of a motile cell were explored by solving Eqs (2) in domains with free boundaries. Note that even though the force-balance equation does not involve time derivatives in and of itself, the coupled system (2) constitutes an initial-value problem and one must specify initial conditions for both variables and the domain, *m*(**x**,0), **u**(**x**,0), and *ω*(0).

To elucidate processes leading to instability of an initially symmetric stationary state of a motile cell and its transitioning to motility, we used initial conditions based on a stationary steady state of the ZV model in a circular geometry *ω*(0) = {(*x*,*y*):*x*^2^+*y*^2^≤1} (such that *a*_0_ = |*ω*(0)| = *π*): **u**(**x**,0) = 0, *m*(**x**,0) = (*μ*_tot_/|*ω*|)(1−*gx*). Note the linear horizontal gradient, added to a steady-state uniform myosin distribution to probe stability of a stationary state; the gradient steepness *g* was assigned values from (0,1]. Note also that given the symmetry of *ω*(0), the definition of *m*(**x**,0) ensures that the solution has a prescribed *μ*_tot_. The initial conditions specified above were used in solving both ZV and ZS models throughout this study.

### Numerical methods

Numerical solutions of the ZV and ZS models were obtained using a generalized version of a mass-conservative algorithm originally developed for solving parabolic equations in moving domains with known kinematics [30]. The method has been shown to converge in space with an order close to 2 in L^2^-norm and ensures exact local mass conservation. The latter is achieved by employing finite-volume spatial discretization [31] and natural neighbor interpolation [32]. The algorithm was developed for modeling cell motility in *Virtual Cell* (VCell), a general-purpose computational framework for modeling cellular phenomena in realistic geometries [33].

To be applicable to a free-boundary problem with the models described above, the original method had to be augmented in several aspects. First, the boundary kinematics is generally not known *a priori* but rather needs to be computed on the basis of the rates that are functions of state variables—the actin velocities, in the ZS model, and the myosin concentrations, in the ZV model. To approximate values of the variables at the points of the boundary where the boundary velocities need to be evaluated, we used the second-order bilinear extrapolation. Once the boundary velocities are obtained, the cell boundary is advanced using a robust front-tracking technique implemented in FronTier, a freely available C++ library for tracking interfaces in two and three dimensions [34]. Accuracy of the algorithm coupled to FronTier was evaluated using several benchmark examples, one of which was based on the models of this study. The tests have shown that the accuracy of the original algorithm is preserved, if in addition to the second-order extrapolation, the front-tracking routine is also at least second-order accurate.

Second, the system (2), consisting of the coupled parabolic and elliptic equations, is nonlinear. Indeed, the equations are coupled via the advection term of the parabolic equation and myosin-dependent stress term in the elliptic equation, as well as through the boundary conditions at the moving boundary; also, the effective transport parameters are functions of the myosin concentration. To solve the system, we implemented a segregated solution strategy [31], in which equations are solved one at a time and nonlinear terms are treated by fixed-point iterations. One advantage of the segregated solver is that it prevents the matrix of a linearized system from becoming very large even with very fine computational grids. The system was advanced in time using an implicit backward Euler time discretization.

For each time step, the segregated method performs fixed-point iterations in two steps. First, we solve for actin velocities using fixed myosin concentrations from the previous iteration. The obtained velocities are then used as a fixed advection field at the next step, where we solve the linearized transport equation for myosin concentrations. Note that at this step, the values of the myosin concentrations in the discretized time derivative correspond to the previous time step, not to the previous fixed-point iteration. At the end of the iteration, maximum absolute differences of two consecutive iterates are checked for convergence. If they are within prescribed tolerances, the iterations stop and the solver reports the velocities and myosin concentrations as the current time step values, otherwise it proceeds to the next iteration and continues until the iterations converge or an imposed maximum for the number of iterations is exceeded. The algorithm is illustrated below for one time step by the mathematical pseudocode, where *m*^*k*^ and **u**^*k*^ are the variable values at the *k*th time step, $m_{n}^{k+1}$ and $u_{n}^{k+1}$ are the *n*th iterates for the (*k*+1)th time step, MaxNumIters is the maximum allowed number of iterations, and ‖⋅‖_∞_ denotes the L^∞^-norm.

set $m_{1}^{k+1}=m^{k}$ and $u_{1}^{k+1}=u^{k}$

for *n* = 1: MaxNumIters

- solve $\alpha \Delta u_{n+1}^{k+1}+\beta \nabla m_{n}^{k+1}-u_{n+1}^{k+1}=0$ to get $u_{n+1}^{k+1}$

- evaluate **u**_eff_ and *d*_eff_ using $u_{n+1}^{k+1}$ and $m_{n}^{k+1}$

- solve $(m_{n+1}^{k+1}-m^{k})/\Delta t=\nabla \cdot \left(d_{\mathrm{eff}}\nabla m_{n+1}^{k+1}-u_{\mathrm{eff}}m_{n+1}^{k+1}\right)$ to get $m_{n+1}^{k+1}$

- calculate absolute errors ${\left\|u_{n+1}^{k+1}-u_{n}^{k+1}\right\|}_{\infty }$ and ${\left\|m_{n+1}^{k+1}-m_{n}^{k+1}\right\|}_{\infty }$

- if solution converged, break the loop, else $m_{n}^{k+1}=m_{n+1}^{k+1}$, $u_{n}^{k+1}=u_{n+1}^{k+1}$

end of segregated loop

if *n*< MaxNumIters $m^{k+1}=m_{n+1}^{k+1}$, $u^{k+1}=u_{n+1}^{k+1}$, else iterations are stagnant.

The segregated solver was validated against a coupled nonlinear solver implemented in COMSOL Multiphysics [35]. Good agreement was observed, with relative differences below 0.3%.

The computations were performed with the following solution parameters: the mesh sizes *h* varied between 0.05 and 0.16, whereas the time step was *Δt* = *ch* with *c* varying from 0.0002 to 0.025 (fast-moving cells required smaller mesh sizes and time steps), the tolerance for the differences of consecutive iterates was 1E-10, and the maximum allowed number of iterations, set at 35, was never reached.

## Results

**Fig 1:**
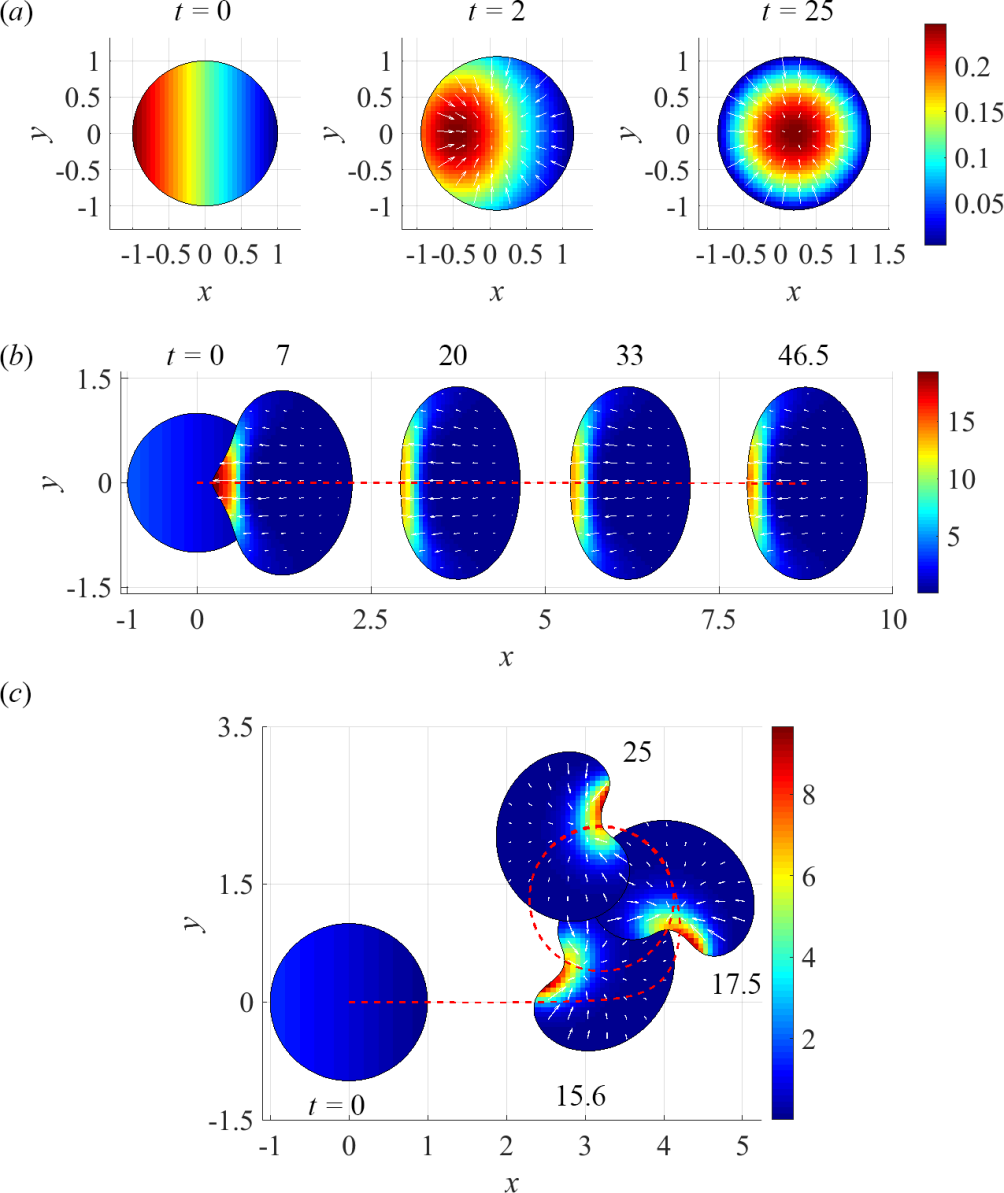
Asymptotically stable mechanical states (in all cases, *α* = 0.5): (*a*) a stationary state of ZS model, (*v*_0_, μ_tot_) = (2.5, 0.125*π*); (*b*) unidirectional translation in ZV model, (*v*_0_, μ_tot_) = (2.5, 2*π*); (*c*) rotations in ZS model, (*v*_0_, μ_tot_) = (2.5, 0.75*π*). Pseudo-colors depict distributions of myosin; arrows are actin velocities; a red dashed line/curve shows the trajectory of a centroid.

The ZV and ZS models were used to simulate transitions of a motile cell from stationary to motile state. For this, as described in section *Initial conditions*, an initially radially symmetric cell was perturbed by superimposing a linear gradient over a uniform distribution of myosin. The emerged steady states fall into three asymptotically stable mechanical modes. For some parameter values, the cell, after a finite displacement, comes to a stop, with a final radially symmetric shape and myosin distribution, indicating the stability of the stationary state (Fig 1*A*). For other parameters, the cell irreversibly breaks symmetry, both in terms of its shape, distribution of myosin, and actin velocity field, and either acquires unidirectional motility (Fig 1*B*) or locks in a rotational mode (Fig 1*C*).

**Fig 2:**
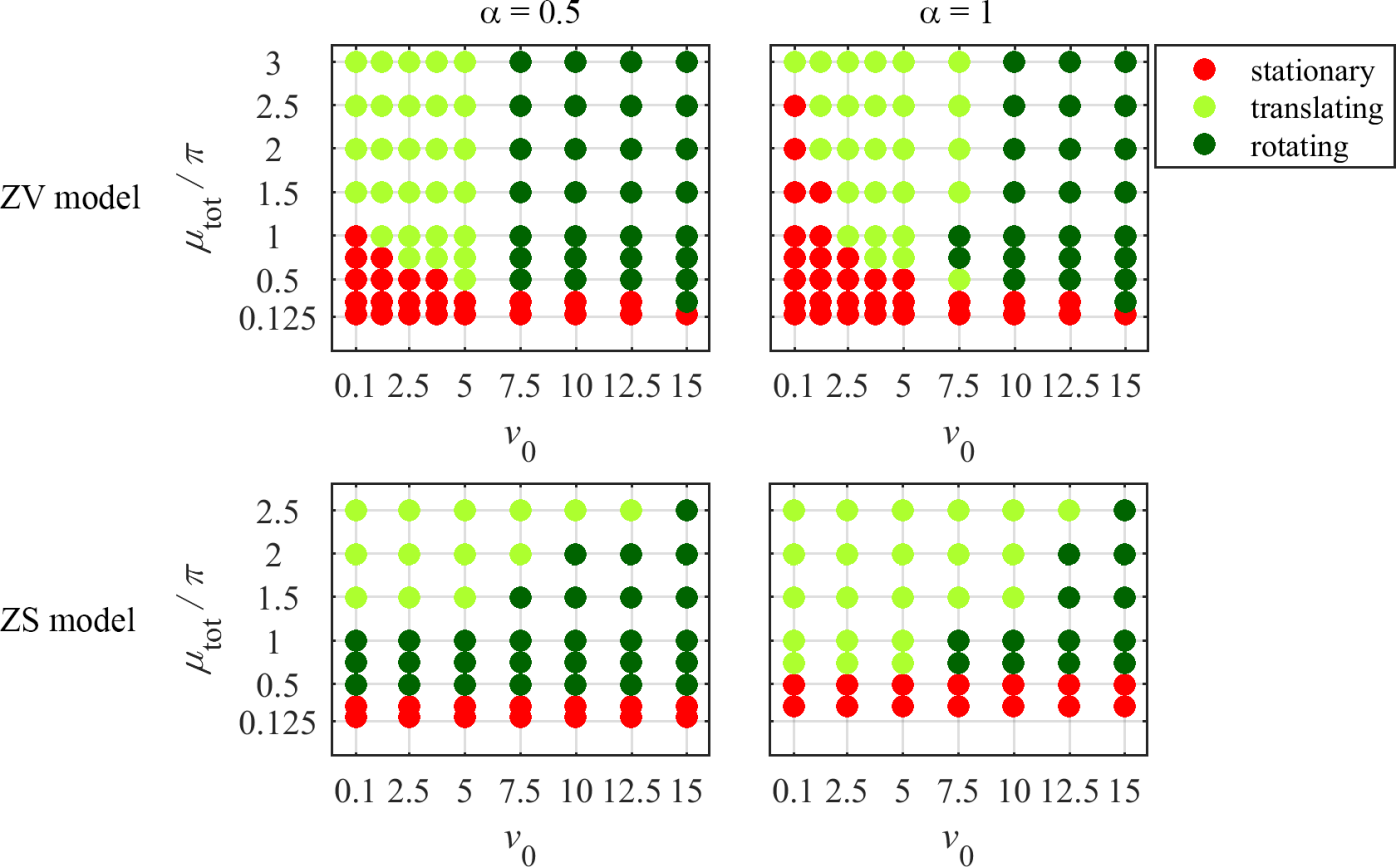
Mechanical states of ZV and ZS models for varying sets (*v*_0_, μ_tot_) and α = 0.5 and 1.

To analyze conditions for transitioning to different types of motility, we scanned the actin growth constant (*v*_0_) and the contractility parameter (*μ*_tot_) for two values of viscosity-adhesion length parameter, *α* = 0.5 and *α* = 1. The values of other model parameters, *β* = 5, *a*_0_ = *π*, *k* = 1.5, *m*_max,*u*_ = 15 and *m*_max,*d*_ = 125, were fixed in all simulations; the choice of these values ensures that the corresponding section of parameter space is representative of various states. The results of parameter scanning are presented in Fig 2 showing cell mechanical states as functions of the model parameters.

It should be noted that distinguishing between translational and rotational modes is sometimes ambiguous, particularly for the states near the borders between the corresponding regions in the parameters space. For example, some states of the ZS model shown in the diagrams of Fig 2 as translating were in fact only ‘piecewise unidirectional’, as the cell in those states would on occasion change its direction. Moreover, in some states in the ZS model, identified as translations, also neighboring the rotations in the diagrams of Fig 2, the cell actually changes its direction but very gradually, so the state could be a rotation with a very long radius. As a practical rule, we labeled states as rotations only if the radius of rotation of the cell centroid was comparable to, or less than, the linear size of the cell.

Below we discuss in detail the conditions required for the straight and rotational motility in our models and the underlying mechanisms.

### Cell becomes motile when myosin contractility is higher than critical

The results in Fig 2 show that cells break symmetry and transition to motility when parameter *μ*_tot_ exceeds a threshold. The threshold behavior originates from a positive feedback between the actin flow and myosin gradients: the contractile forces, generating the centripetal flow of myosin, are proportional to the myosin gradients, which, in turn, are reinforced by the advection of myosin. This positive feedback results in steep myosin gradients and, potentially, symmetry breaking, but below a critical value of *μ*_tot_, these gradients are prevented by dissipative viscous forces and myosin diffusion, and the cell remains stationary and radially symmetric. Above the critical value of *μ*_tot_, steep gradients of myosin are developed and the radially symmetric stationary state becomes unstable. While kinematics of a free boundary plays a key role in the symmetry breaking and transitioning to motility in both models, the loss of symmetry in the ZV model also occurs in fixed domains. In the next section, we discuss effects of the boundary conditions for actin flow on solutions in domains with fixed and free boundaries.

Linear stability analysis can be used to estimate critical values of *μ*_tot_ in a simplified ZV model in a fixed domain. Consider a 1D ZV model on the fixed-length segment *∂ω* = {*x*∈(0,1)} in the limit *m*_max,1_, *m*_max,2_ → ∞, so that *d*_eff_ = 1 and *u*_eff_ = *u*(*x*,*t*). Then, Eqs (2) become *αu*_*xx*_ + *βm*_*x*_−*u* = 0, *m*_*t*_ = *m*_*xx*_−(*um*)_*x*_. In this model, varying the contractility constant *β* is equivalent to rescaling *μ*_tot_. Indeed, *β* could be excluded altogether by renormalizing *m*: $\tilde{m}=\beta m$, but in what follows parameter *β* is kept for generality. The symmetric steady state is characterized by uniform myosin distribution and absence of actin flow, *u* = 0, *m* = *μ*_tot_. Its stability is probed by imposing small perturbations, *δu*(*x*,*t*) = *δu*_0_ exp(*λt*+*iqx*), *δm*(*x*,*t*) = *δm*_0_ exp(*λt*+*iqx*) with 0<*δu*_0_<<1, 0<*δm*_0_<<1 and *q* = *π*, 2*π*,…, so that *u* = *δu* and *m* = *μ*_tot_+ *δm*. The perturbations satisfy the linearized system of differential equations, *αδu*_*xx*_+*βδm*_*x*_−*δu* = 0, *δm*_*t*_ = *δm*_*xx*_−*μ*_tot_(*δu*)_*x*_, and the corresponding system of linear algebraic equations, −(1+*αq*^2^)*δu*_0_+*iqβδm*_0_ = 0, *iqμ*_tot_*δu*_0_+(λ+*q*^2^)*δm*_0_ = 0, yields nontrivial solutions for *δu*_0_ and *δm*_0_, if *λ*(*q*) = *q*^2^(*βμ*_tot_(1+*αq*^2^)^−1^−1). The perturbations grow if *λ*(*q*)>0, with the fastest growing mode having the minimal wave number, *q*_min_ = *π*, and thus involving a large-scale redistribution of myosin. Thus, the symmetric state becomes unstable if *βμ*_tot_>1+*π*^2^*α* or, in the dimensional form, *σM*_0_*πL*^2^>*D*(*ξL*^2^+*π*^2^*η*).

**Fig 3:**
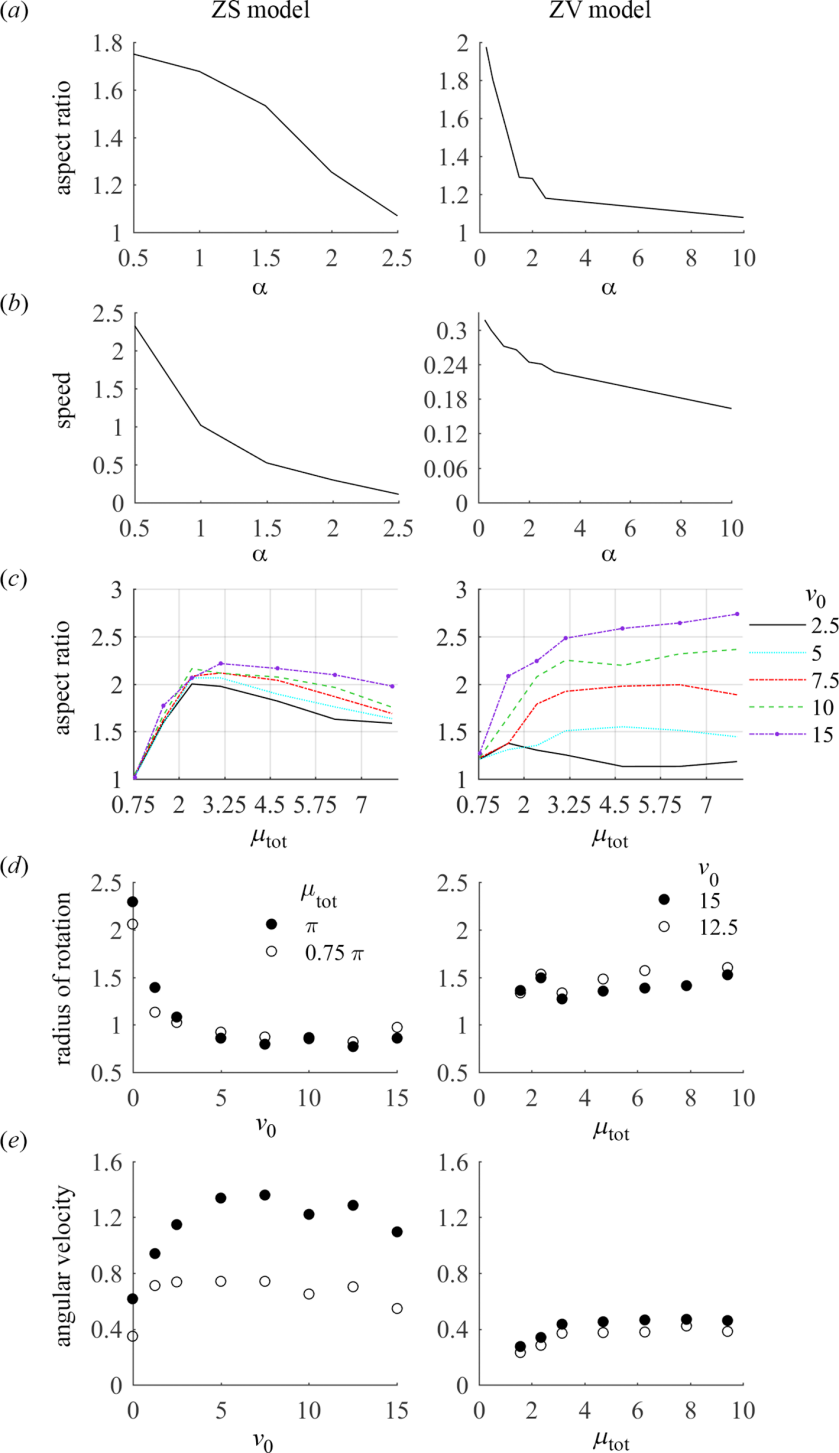
Aspect ratios and translational or linear rotational speeds of steadily moving cells. (*a*) Aspect ratio as a function of viscosity-adhesion length α; the results were obtained with (*v*_0_, μ_tot_) = (2.5, 1.5π) for ZS model, and with (*v*_0_, μ_tot_) = (5, 1.5π) for ZV model; aspect ratios were computed as ratios of the longest to shortest distances between cell boundary and cell centroid. (*b*) Dimensionless translational or linear rotational speed of a cell centroid as a function of viscosity-adhesion length α; model parameters are as in panel (*a*). (*c*) Aspect ratio as a function of *v*_0_ and μ_tot_; the results were obtained with α = 1 for ZV model and with α = 0.5 for ZS model. (*d*) Radius of rotation of a cell centroid as a function of *v*_0_ and μ_tot_, with values of α as in (c). (*e*) Dimensionless angular velocity of a cell centroid as a function of *v*_0_ and μ_tot_, with values of α as in (c).

The instability criterion predicts that the critical value of *βμ*_tot_ is an increasing function of *α*. The results of Fig 2 indicate that this prediction, while obtained by analyzing a ZV model in a fixed domain, holds for the free-boundary models as well. Indeed, the competition between the myosin contractile stress and dissipative processes, mathematically expressed in the instability condition, drives the initiation of motility in the free-boundary models. As described at the beginning of this section, the transition to motility occurs when the contractility, reinforced by the model positive feedback, prevails over the dissipation. For *α*≥1, the dimensional form of the instability criterion reduces to *σM*_0_*L*^2^>*πDη*: the total myosin stress needs to overcome the smoothing effects of actin viscosity and myosin diffusion, while the adhesion strength does not matter. In the limit of large values of *α*, the actin network is effectively stiff and thus does not allow for significant actin flows, which makes the cell more symmetric and as a consequence less motile and slower. Our simulations confirm this prediction (Fig 3A and 3B). In the opposite limit of highly deformable actin network, *α*<<1, the instability criterion reduces to *σM*_0_>*ξD*, so the cell becomes motile if the characteristic myosin stress is able to generate actin flow that overcomes myosin diffusion, which requires the weakening of adhesions and strengthening of myosin, in agreement with the experiment [7].

Finally, it is worth noting that whereas the motility threshold in the ZS model is independent of *v*_0_, the critical values of *μ*_tot_ in the ZV model, where the actin growth is affected by myosin, vary with *v*_0_ (Fig 2). Indeed, the cell described by the ZV model with *v*_0_ = 0 would not transition to motility for any *μ*_tot_, because in this case, the myosin influence on the boundary is lost. Therefore, in this version of the model motility initiates only for finite values of *v*_0_, which drop with the increase of *μ*_tot_. In the ZS model, the cell with a sufficiently high *μ*_tot_ initiates motility even as *v*_0_→0, because the asymmetric myosin pulls the boundary inward asymmetrically, and the area-preserving term causes the effective protrusion.

### Shape and speed of the motile cell

**Fig 4:**
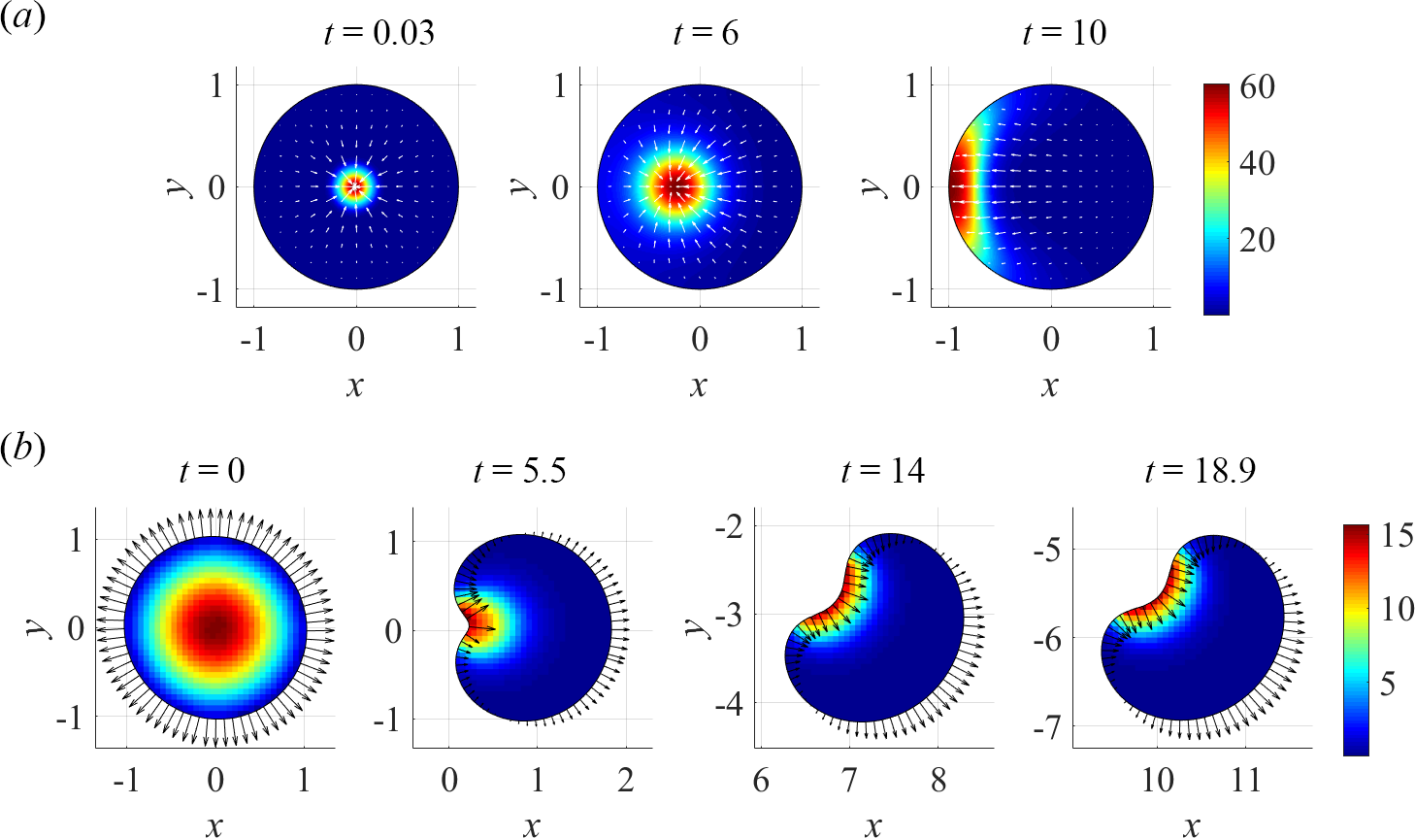
Symmetry breaking in a fixed circle and in a free-boundary problem. (*a*) Instability of a symmetric steady state of ZV model in a fixed circle: snapshots of dimensionless myosin density (pseudo-color) and actin velocities (arrows) at specified times *t* after myosin was slightly shifted left of center; computations were done for μ_tot_ = 1.5π and α = 0.5. (*b*) Transition to unidirectional motility in ZS model; dimensionless myosin concentration (pseudo-colors) and boundary velocities (arrows) are shown for the solution obtained with α = 1, *v*_0_ = 5, and μ_tot_ = 1.5π; the cell assumes steady unidirectional motility after *t* = 14 (S2 Movie).

Similar to the 1D ZV model analyzed in the previous section, the symmetric state of the 2D version of the ZV model becomes unstable for sufficiently high *μ*_tot_ even in a fixed geometry, with myosin relocating to the cell boundary. Fig 4A illustrates the instability of the radially symmetric steady state, in which the maximum of myosin was slightly shifted to the left of the cell center. Qualitatively, because of the zero actin velocities at the boundary, a second, initially small, local maximum of myosin appears at the boundary point closest the main maximum due to slightly faster diffusion. The competition between the two maxima lowers the myosin gradients on the left side of the main one, resulting in a net force acting on it in the left direction. Hence, the relocation of myosin to the left segment of the boundary. In the cell with a free boundary, the redistribution of myosin is conferred to boundary velocity, resulting in slower outward and eventually inward movements of the part of the boundary that becomes the cell rear. The cell movement further skews the myosin towards the rear. For the small to moderate rate of actin growth and cell speeds, the cell maintains a convex shape and a steady unidirectional motion, with myosin forming a wide band at the rear edge (Fig 1*B* and S1 Movie).

In the ZS model of a fixed symmetric cell, an inward actin flow at the membrane prevents myosin from accumulating there. As a result, the symmetric solution with a myosin maximum at the center remains stable even for *μ*_tot_ above the threshold. Indeed, shifting the maximum from the center in this case increases the myosin gradients and centripetal forces on the ‘shorter’ side and decreases them on the ‘longer’ side, netting a stabilizing force. In the free-boundary problem, however, the symmetric solution, stable at low *μ*_tot_ (Fig 1*A*), becomes unstable above the motility threshold. Fig 4B and S2 Movie illustrate a transition to unidirectional motility that occurs in the ZS model with sufficiently large *μ*_tot_ and small to moderate *v*_0_. As the myosin cluster shifts slightly from the cell center, the closer side is pulled inward faster and becomes the prospective cell rear, while the opposite side, where the protrusions are faster than retractions, becomes the cell front. In the ensued motility, myosin is pressed against the rear and in turn exerts a stronger inward force on the proximal portion of the boundary, developing a local concavity. If the movement is sufficiently fast, the myosin spreads along the portion of the boundary with negative curvature. This positive feedback between the myosin asymmetry, actin flow, and cell movement is the key to the stable motility.

Our simulations show that the aspect ratio of a steadily moving cell varies between 1 and 3 (Fig 3C), in agreement with experimental observations [5]. Note that in contrast to the ZV model, where the cell aspect ratio grows moderately with *v*_0_, it becomes essentially independent of model parameters in the ZS model with *μ*_tot_/*π*>1. This can be qualitatively understood by noting that myosin, which in a moving cell accumulates in the middle of the rear, exerts comparable forces on the front and side portions of the boundary. Then, because the myosin-generated flow decreases with distance at similar rates in all directions, the distances from the rear to the front and the sides should be on the same order, yielding the average aspect ratio ~ 2.

**Fig 5:**
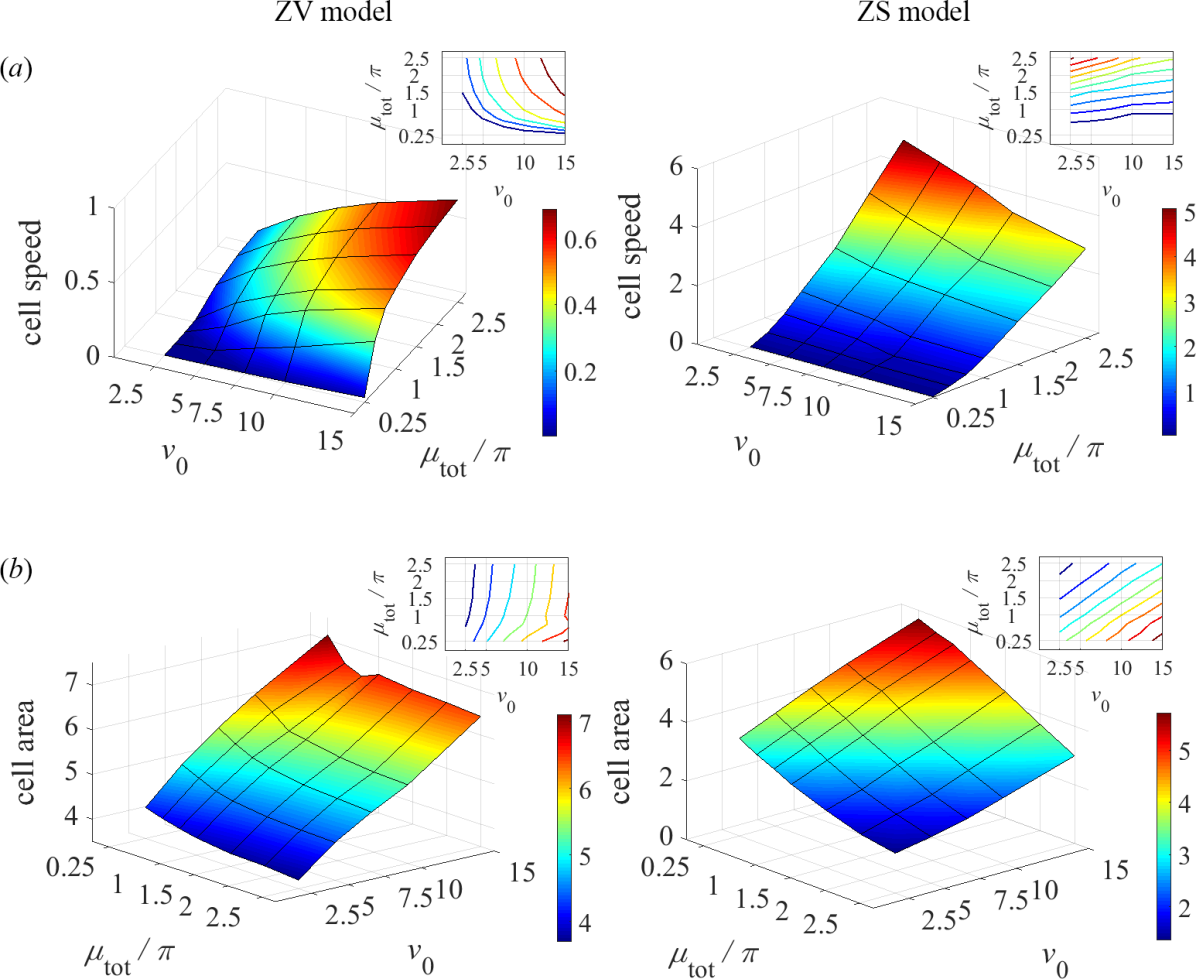
Cell speeds and areas as functions of *v*_0_ and μ_tot_. (*a*) Dimensionless translational or linear rotational speed of a cell centroid were obtained with α = 1 for ZV model and α = 0.5 for ZS model. (*b*) Dimensionless steady-state areas for values of α as in panel (*a*). Insets in both panels: corresponding level sets.

The ZS model generally predicts significantly higher cell speeds compared to those in the ZV model (Fig 5A). This is because in the ZS model, the fast centripetal flows generated by myosin at the rear boundary tend to decrease the cell area, leading to fast effective protrusion at the front, as actin can grow rapidly against the lowered membrane tension. As a result, the cell speed increases but the cell area decreases with total myosin (Fig 5B). Interestingly, the cell speed in the ZS model decreases slightly with the actin growth rate *v*_0_, which can be understood by noting that the cell area in this model increases with *v*_0_, thus mitigating the effect of myosin. In the ZV model, the cell speed is virtually insensitive to *μ*_tot_, for *μ*_tot_>*π*, because the term with *v*_0_ in the expression for *v*_p_ becomes inessential for *m*∼*μ*_tot_/*π*>1. For the same reason, the cell area is also insensitive to *μ*_tot_.

Importantly, our model predicts that there are no short-wavelength instabilities in the cell shape (like fingering instabilities characteristic for some physical free-boundary models), which is supported by the experiment: there are small fluctuations on the experimentally observed cell boundaries, but they mostly do not grow.

### Mechanics of the straight and turning motility

The most nontrivial and important property of our models is that they predict rotational states with a radius of rotation comparable to the cell size in large regions of their parameter space (Fig 2). Note that both the radius of rotation and the angular velocity are not particularly sensitive to parameter values (Fig 3D and 3E).

**Fig 6:**
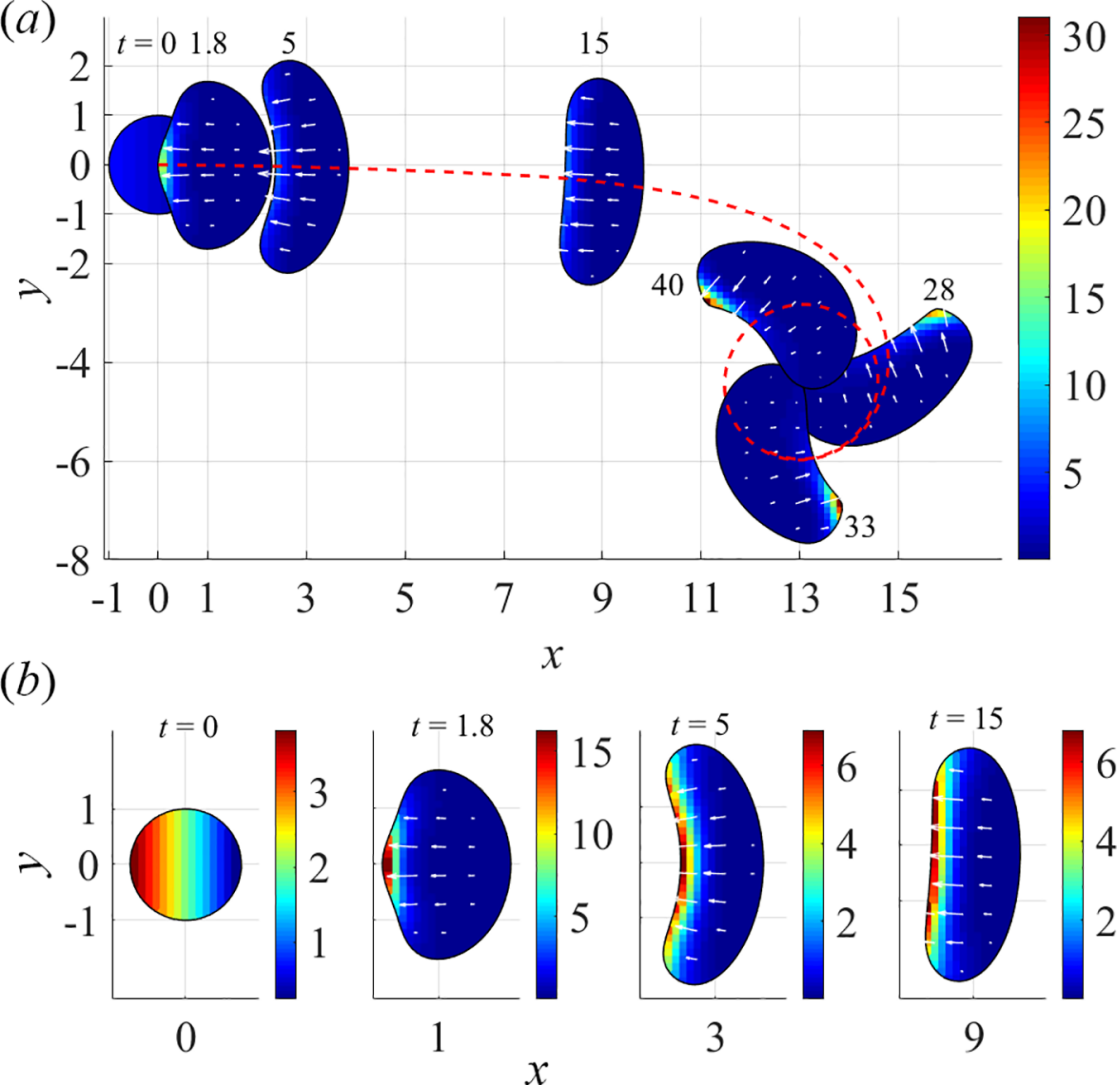
Onset of steady rotations in ZV model, (*v*_0_, μ_tot_, α) = (12.5, 2π, 1). (*a*) Entire cell trajectory and cell centroid track (red dashed curve). (*b*) Snapshots of transient myosin distributions with individual color scales during a transient, and with white arrows representing actin velocities (S3 Movie).

Emergence of cell turning in the models can be qualitatively understood by analyzing the loss of stability of a planar axial symmetry characteristic of the straight moving cell. In the ZV model, the steady rotations are observed for *v*_0_ exceeding a threshold that is largely insensitive to either *α* or *μ*_tot_. Fig 6 and S3 Movie illustrate, for a particular parameter set, how rotations come about in the ZV model during a transient movement following a ‘nudge’ applied to a stationary cell in the form of an initial horizontal gradient of myosin. The initial convex cell shape is favorable for maintaining a unipolar axially symmetric myosin distribution, and the resulting motility is unidirectional. But due to sufficiently large *v*_0_, fast boundary velocities at cell’s sides tend to elongate the cell in *y*-direction, making it prone to developing a concavity at the cell rear. In such a shape, the myosin spreads along the rear part of the boundary more uniformly (Fig 6*B*, *t* = 5), a distribution that is no longer stable. Indeed, even a slight asymmetry in the distribution of myosin, reinforced by the positive feedback from actin velocities and myosin accumulation due to faster movement of the corresponding portion of the rear boundary, breaks the axial symmetry (Fig 6*B*, *t* = 15). As a result, a stable asymmetric cell shape emerges as the cell locks in rotations (Fig 6*A*), with myosin aggregated at a high-curvature portion of the boundary.

While the same mechanisms underlie the turning behavior in the ZS model, the two models yield significantly different results for the parameter regions of rotations. This is due to the differences in boundary conditions that reflect the opposing assumptions about the strength of adhesions at the cell periphery, and in ways of conferring the myosin dynamics onto kinematics of the boundary. Unlike the ZV model, the *v*_0_ threshold for rotations in the ZS model strongly depends on *μ*_tot_ and *α* (Fig 2). In particular, the rotational states may exist for any *v*_0_, if *α* is sufficiently small. Note also, that the concavity of the cell shape does not always destabilize unidirectional motility in the ZS model (Fig 4B).

**Fig 7:**
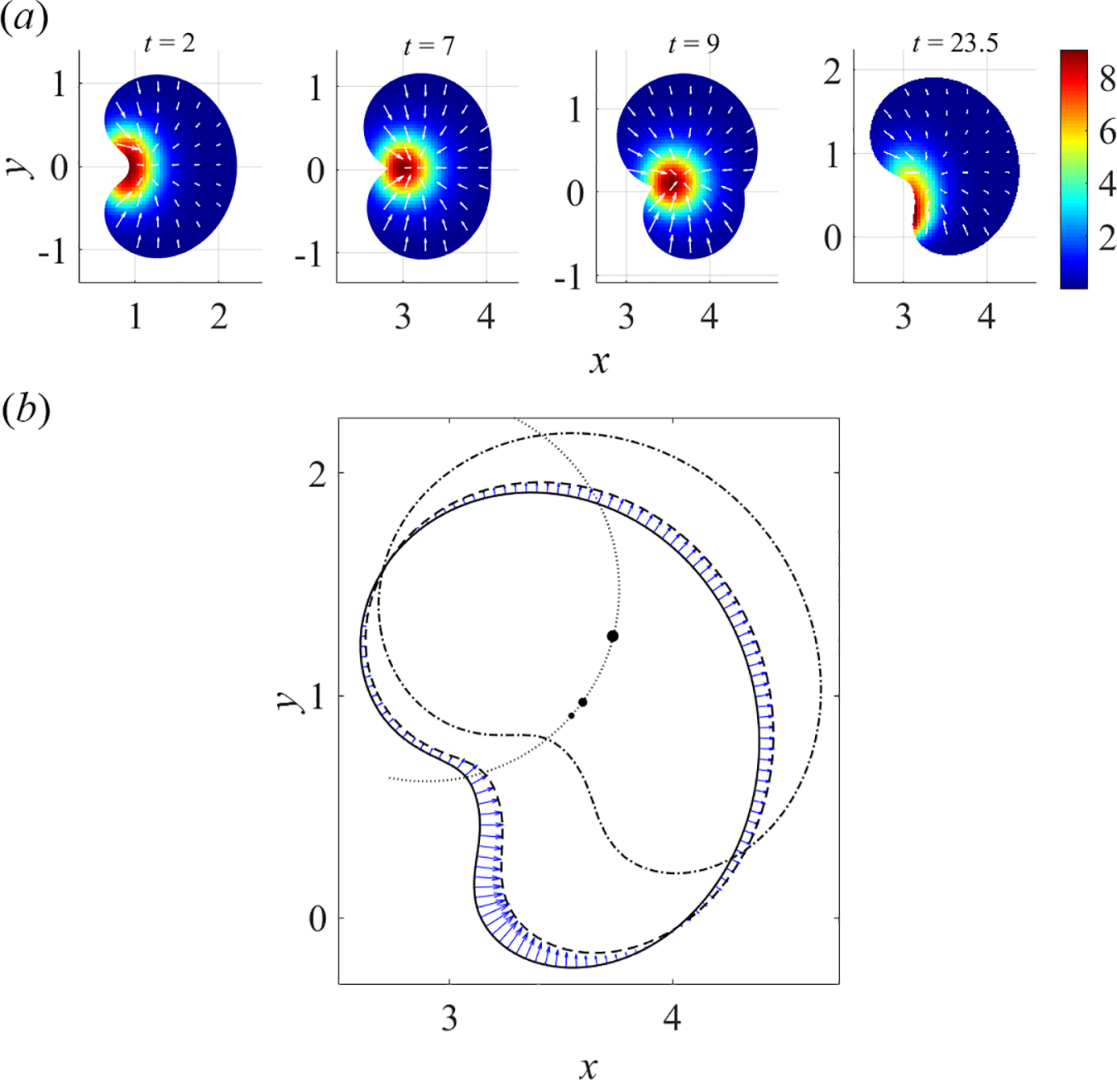
Steady rotations in ZS model, (*v*_0_, μ_tot_, α) = (2.5, 0.75π, 0.5) (see also S4 Movie). (*a*) Transient distributions of myosin (pseudo-colors) and actin velocities (arrows): *t* = 2, an initially symmetric cell with centroid at (*x*, *y*) = (0,0) self-polarizes and assumes fast unidirectional motility, myosin accumulates in a semi-circular band, pulling the rear inwards to form a ‘dip’; *t* = 7, the cell slows down and becomes unstable, as myosin is now close enough to cell front to be able to pull it in as well; *t* = 9, loss of axial symmetry, as the lower part of the cell with steeper myosin gradients is pulled inwards faster than the upper one; *t* = 23.5: emergence of stable asymmetric myosin distribution and cell shape, as the cell locks in rotations (see Fig 1*C*). (*b*) Cell shape and boundary velocities in steady rotations. Positions of the cell boundary and centroid at *t* = 23.5, 23.6, and 24 (solid, dashed, and dotted-dashed contours, respectively, and filled circles with larger size corresponding to later time). Faster boundary velocities (arrows) in the high curvature region, consistent with the location of steep myosin gradients (panel (*a*)), ensure rotational motility with a circular trajectory of the centroid (dotted arc), see also Fig 1*C*.

Fig 7 and S4 Movie illustrate the onset of turning in ZS model. If the contractility due to myosin is strong, myosin forms a radially symmetric aggregate, which in a translating cell is skewed to the cell rear, pulling the rear boundary inward and maintaining the cell propulsion. When the myosin aggregate is sufficiently close to the rear boundary, it pulls the center of the cell rear inward stronger than the sides of the rear edge, creating a ‘dip’ at the center of the rear edge and giving the cell a characteristic keratocyte fan-like shape, in which the sides of the cell lag behind the center. For the parameters in the upper left corner of the parameter space (Fig 2), the cell motion is fast, and in the frame of the cell, myosin is effectively swept towards the rear and ‘pressed’ against the rear boundary; in these conditions, the translational motility remains stable (Fig 4B). For intermediate values of *μ*_tot_, the cell moves slower and the myosin aggregate maintains its radial symmetry and remains close to the cell centroid (Fig 7*A*, *t* = 7). In this position, myosin is able to pull inward not only the rear but also the front of the cell, making the axial symmetry of the system unstable. Indeed, even a slight random asymmetry in either the myosin distribution or the cell shape induces and reinforces the asymmetry of the other. If, for example, the myosin aggregate becomes slightly closer to one side, this side is pulled inward faster than the other, which brings even more myosin to the side that is pulled inward, because the shift of that side effectively sweeps myosin towards it (Fig 7*A*, *t* = 9).

Once the axial symmetry of cell shape and the position of the myosin aggregate is broken (Fig 7*A*, *t* = 23.5), the boundary velocity field becomes asymmetric as well (Fig 7*B*). It is then clear that a steady movement of a cell with an asymmetric shape and asymmetric boundary velocities (where faster displacements occur at the location of higher myosin gradients) must involve rotations. Indeed, by connecting consecutively the ends of the arrows representing normal displacements of points of the boundary in Fig 7*B*, one recovers the same contour in a rotated position, as the centroids of the cell in different positions belong to the same circle (Fig 7*B*). We also note that the shape of the expanding portion of the cell boundary is reminiscent of spirals described by more abstract models of rotating free boundaries [36].

## Discussion

In this paper we systematically explored the ability of a minimal actin-myosin contractility model [7, 14] to reproduce observed mechanical states of the simplest motile cell. The model analysis has shown that the mechanical state of the cell critically depends on just three dimensionless parameters representing the myosin contractility, characteristic viscosity-adhesion length, and actin growth. For the large viscosity-adhesion length, the actin network becomes effectively stiff and does not allow for significant actin flows, which makes the cell more symmetric and as a consequence less motile and slower, and in the limit of very large values of viscosity-adhesion lengths, the cell is stationary. In the opposite limit of short viscosity-adhesion lengths, myosin forms a very small high-density aggregate, which affects the actin network only locally. In this regime, the steady motility is impossible, and the cell starts to pivot. Thus, an important conclusion is that to move straight and steadily, the cell has to keep the viscosity-adhesion length on the order of unity (to adjust the ratio of actin viscosity to adhesion strength so that it is on the order of the cell area). Interestingly, this conclusion is consistent with estimates based on the experimental data for keratocytes [5].

Intuitively, if myosin contractility is weak, the myosin spreads uniformly and the cell remains stationary and symmetric. Above a contractility threshold, the cells become motile. The mode of motility depends on the boundary conditions. For the zero actin velocity at the boundary and the sufficiently small actin growth constant and cell speed, the convex-shaped cell maintains unidirectional motility, with myosin concentrated in a band at the rear edge. With the rate of actin growth above a certain threshold, the increase of the cell speed is sufficient for the cell to lose its planar axial symmetry and start rotating. With the zero-stress boundary conditions, rotations occur for intermediate contractility strengths, whereas in the high contractility range, the fast cells stabilize their unidirectional movement, as myosin being effectively compressed into a long band at the rear edge. We found that both explored boundary conditions explain general features of the keratocyte motility, but there are interesting differences in the predicted behaviors, as discussed above.

The main finding of our study is that the contractile mechanism of motility results in a very robust turning behavior of the cell: in the models with both explored boundary conditions, the cell moving along a circular trajectory is not an anomaly but rather a solution that exists in a large region of the model parameter space. Broadly speaking, the cell starts turning in conditions of breaking the planar axial symmetry of its myosin distribution; in the ZV model the transition to rotation is controlled by the rate of actin growth, whereas in the ZS model–by all three independent model parameters. Turning motile behavior is an important part of the cell mechanical response in chemotaxis [37] and galvanotaxis [38], and our model generates intuition about the turning mechanism.

One important test of our model is that the solutions exhibit a characteristic fan-like keratocyte shape, with the side-to-side distance greater than the front-to-rear distance and aspect ratio between 1 and 3, in excellent agreement with the observations [5]. Moreover, the predicted aspect ratio in the ZS version is nontrivial and biphasic, reaching a maximum at intermediate myosin contractility and decreasing at very weak or strong contractility, indeed observed in [5]. Similarly, the model predicts that the lamellipodial area increases at higher adhesion and lower myosin contractility, and that the cell speed increases with myosin contractility, as observed [5]. Lastly, in agreement with the experiment [7], higher myosin contractility and/or lower adhesion strength are predicted to promote the cell polarization and motility initiation. One significant prediction of our model is that both self-polarization of the cell and its turning behavior can, in principle, occur, without complex adhesion dynamics. While it was observed that a nonlinear stick-slip adhesion behavior accompanied cell polarization [7], it remains an open question whether this nonlinear behavior is essential.

The model predicts that the stable motile behavior of the cell requires tight regulation of the total lamellipodial area. We hypothesized that this regulation is mechanical, through the membrane tension. Indeed, perturbations of the total membrane area and membrane tension were found to change the lamellipodial area in a predictable way, and drastic perturbations destabilized the cell [29]. We find that cell polarization may not depend on the cell ability to move: in the ZV model, myosin distribution and actin flow become asymmetric even in the stationary symmetric domain. However, for the motility initiation, protrusion of the boundary is obviously essential (note that while motility in the ZS free-boundary model with sufficiently high *μ*_tot_ can be initiated even with *v*_0_→0, the area-preserving term of *V*_p_ in this limit effectively induces protrusion of the front, which is less affected by myosin, see sections *Model* and *Cell becomes motile when myosin contractility is higher than critical*).

Keratocyte motility and especially the peculiar and steady shape of the moving cell inspired a great deal of free-boundary modeling in the past decade. Our model is based on the well-justified assumption that the mechanical force balance determines cell shape and movements. Conceptually, our model is similar to the active gel models [24, 39], originating in the soft-matter physics. Some models were based on the viable idea that certain self-organized chemical patterns are upstream from the actin-myosin machinery [22, 40, 41], but majority of studies explored mechanical models [42–44]. A variety of numerical techniques–Potts models [40, 45], phase-field method [42, 44], immersed boundary method [43]–were used in respective simulations. Keratocyte polarization was modeled in [7, 46]. Alternative turning mechanisms, very different from the one predicted by our model, were computationally explored in [47, 48]. The fact that the majority of the models reproduce the keratocyte shapes and motile behavior corroborates the existing biological intuition about the keratocyte lamellipodium as the most basic, streamlined and robust actin-myosin motile structure [49]. Each of the cited studies added invaluable insights to understanding multifaceted aspects of cell motility; a relative advantage of our model is in that it is most easily connected to the experimentally observed biophysics of force balance and myosin transport in keratocytes [5, 7].

**Fig 8:**
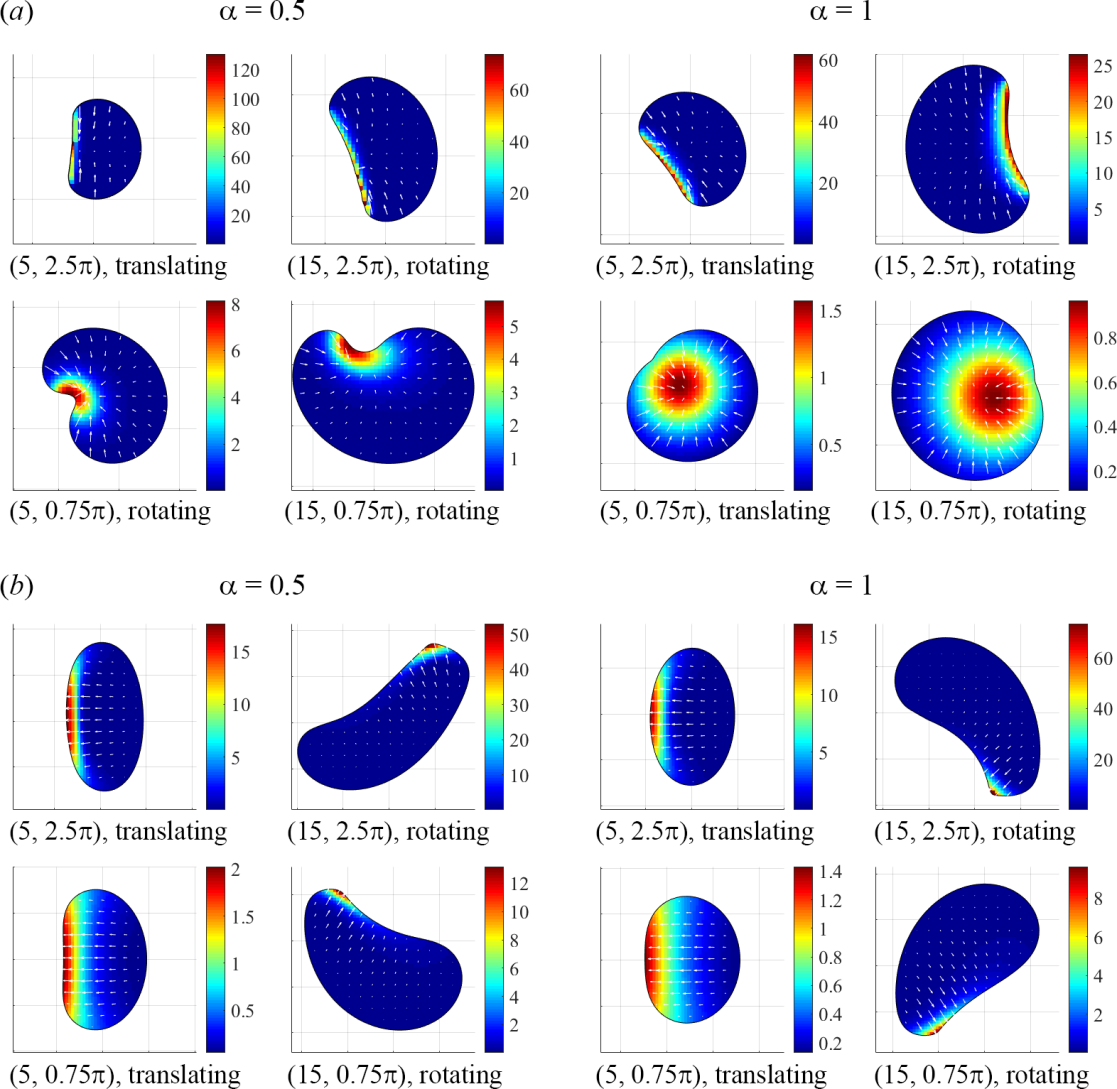
Steady-state cell shapes, myosin distributions (pseudo-colors), actin velocities (arrows), and motility types from solutions of the ZS (*a*) and ZV (*b*) models obtained for specified parameter values (*v*_0_, μ_tot_). Gridlines are spaced uniformly with *h* = 1.

The minimal model we explored already predicts a wealth of motile behaviors (Fig 8). It is known, however, that even the cell as streamlined for locomotion as keratocyte has complexities that far exceed our minimal model. The two main aspects that need to be added to the model to make it more realistic are: spatially graded actin polymerization independent of myosin [10] and dynamic nonlinear adhesions. Complex effects of dynamic and non-homogeneous adhesions already attracted special attention and were simulated in [7, 46, 50]. It will also be interesting to explore how the predicted cell dynamics depend on actin density [51], more complex constitutive relations for the actin-myosin stress [52], membrane curvature [53, 54], elastic [55] and anisotropic [49] effects in the actin network. Our minimal free-boundary model might be useful for future modeling of other modes of cell motility [56] and collective cell movements [57, 58]. Lastly, for decades, research focused on understanding cell movements on flat 2D surfaces, and only recently exploration of cell crawling through three-dimensional (3D) matrices, more physiologically relevant, has begun experimentally [59] and theoretically [60–62]. Extension of our model to 3D will be a challenging, yet necessary, effort.

## Supporting information

S1 Appendix Variations of the model. Supplemental text explaining effects of various model assumptions, equations and boundary conditions, including explicit consideration of actin dynamics and more detailed model of myosin transport.
(DOCX)

S1 Movie Example of steadily moving cell predicted by simulations of the zero velocity model. (AVI)

S2 Movie Example of steadily moving cell predicted by simulations of the zero stress model. (AVI)

S3 Movie Example of rotating cell predicted by simulations of the zero velocity model. (AVI)

S4 Movie Example of rotating cell predicted by simulations of the zero stress model. (AVI)
