# Supplementary material for: A free-boundary model of a motile cell explains turning behavior
Source: PLoS Comput Biol. 2017 Nov 14;13(11):e1005862. doi: 10.1371/journal.pcbi.1005862 (PMC5705165; doi:10.1371/journal.pcbi.1005862)
Supplement: S1 Appendix — Supplemental text explaining effects of various model assumptions, equations and boundary conditions, including explicit consideration of actin dynamics and more detailed model of myosin transport. (DOCX) [file pcbi.1005862.s001.docx]

***S1 Appendix: variations of the model.***

***2D character of the model and cytosolic pressure.***

The most natural way to investigate the role of pressure in mechanics model of cell motility is to start with inclusion of mechanics of cytosol into the model. Most of research was done by using the so-called two-phase poroviscous theory of the cytoplasm, which treats the cytoskeleton and cytosol as two interpenetrative viscous fluids (Drew and Segel, 1971; Dembo and Harlow, 1986; Kuusela and Alt, 2009; Cogan and Guy, 2010). Our formulation here is close to that of (Oliver et al., 2005), but note that a number of constitutive relations and assumptions that we use are original. Characteristic poroviscous theory consists of the mass conservation equations for the network and for the cytosol:

$\underbrace{\frac{\partial\Phi}{\partial T}+\nabla\cdot\left(\Phi\mathbf{U}_{n}\right)}_{\begin{matrix} \text{rate of change of network} \\ \text{ volume fraction} \end{matrix}}=\underbrace{-J}_{\begin{matrix} \text{net rate of} \\ \text{assembly/} \\ \text{disassembly} \end{matrix}},\underbrace{\frac{\partial\left(1-\Phi\right)}{\partial T}+\nabla\cdot\left(\left(1-\Phi\right)\mathbf{U}_{s}\right)}_{\begin{matrix} \text{rate of change of cytosol} \\ \text{ volume fraction} \end{matrix}}=\underbrace{+J}_{\begin{matrix} \text{net rate of} \\ \text{assembly/} \\ \text{disassembly} \end{matrix}},J=r\left(\Phi-\Phi_{0}\right)$, (Eq S1)

and of the force balance equations for the network,

$+\underbrace{\eta\nabla^{2}\mathbf{U}_{n}}_{\text{gel viscosity}}+\underbrace{\sigma\nabla M}_{\begin{matrix} \text{myosin con-} \\ \text{tractile stress} \end{matrix}}\underbrace{-\Phi\nabla P}_{\text{pressure}}=\underbrace{K\left(\mathbf{U}_{n}-\mathbf{U}_{s}\right)}_{\begin{matrix} \text{network-cytosol} \\ \text{ drag} \end{matrix}}+\underbrace{\xi\mathbf{U}_{n}}_{\begin{matrix} \text{adhesion} \\ \text{ drag} \end{matrix}},$ (Eq S2)

and for the cytosol,

$\underbrace{\left(1-\Phi\right)\nabla P}_{\text{pressure}}=\underbrace{K\left(\mathbf{U}_{n}-\mathbf{U}_{s}\right)}_{\begin{matrix} \text{network-cytosol} \\ \text{ drag} \end{matrix}}$. (Eq S3)

We also add the transport equation for myosin:

$\frac{\partial M}{\partial T}=\underbrace{D\nabla^{2}M}_{\begin{matrix} \text{ myosin } \\ \text{diffusion} \end{matrix}}-\underbrace{\nabla\cdot\left(\mathbf{U}_{n}M\right)}_{\begin{matrix} \text{myosin drift with } \\ \text{ actin flow} \end{matrix}}$. (Eq S4)

Here the model dependent variables are as follows:$\mathbf{U}_{n}=\left(U_{n},V_{n},W_{n}\right),\mathbf{U}_{s}=\left(U_{s},V_{s},W_{s}\right)$are the velocities of the cytoskeletal network and cytosol, respectively;$\Phi$is the network volume fraction;$P$is the cytosol’s pressure;$M$is the myosin density. The independent variables are time$T$and spatial coordinate$\mathbf{X}=\left(X,Y,Z\right)$. Note that we formulate the theory in 3D. The model parameters include$r$, the rate of actin turnover;$\Phi_{0}$, the equilibrium volume fraction of the network;$\eta$, the network viscosity;$\sigma$, the myosin strength;$K$, the hydraulic resistance (network-solution drag) coefficient;$\xi$, the network-surface drag coefficient;$D$, the effective myosin diffusion coefficient. For simplicity, we neglect the so-called solvation and swelling pressures, which in principle can be equal to zero (Oliver et al., 2005). Note that all these parameters can be functions of$\Phi$, as well as functions of$\mathbf{X}$and$T$.

The cell geometry is described by the ventral surface at$Z=0$and dorsal surface at$Z=H\left(X,Y,T\right)$. Boundary conditions include zero flux conditions at both boundaries for the mass conservation equations. Kinematic conditions are: at the ventral surface ($Z=0$):$W_{n}=W_{s}=0$; at the dorsal surface ($Z=H\left(X,Y,T\right)$):$\mathbf{U}_{n}\cdot\mathbf{n}=\mathbf{U}_{s}\cdot\mathbf{n}$, where$\mathbf{n}$is the outward unit normal vector. There are slip conditions for tangential component of all velocities at both ventral and dorsal surfaces. Finally, there is the force balance condition at the dorsal surface:

$-\left(1-\Phi\right)P\mathbf{n}+\Phi\eta\nabla\mathbf{U}_{n}\cdot\mathbf{n}=\gamma\kappa\mathbf{n}$. (Eq S5)

Here$\gamma$is the constant isotropic membrane tension, and$\kappa$is twice the mean curvature;$\kappa<0$if the dorsal surface is convex. We also assume that there is no myosin near the dorsal surface.

In addition to the model assumptions described in the main text concerning the forms of the network viscous stress, myosin contractile stress and simplified myosin transport equations, and to the standard assumptions of the two-phase poroviscous theory (Oliver et al., 2005; Cogan and Guy 2010), the main assumptions that are behind system of equations (S1)-(S5) are as follows. We assumed that the connection points between the adhesive molecular chains and actin filaments are distributed uniformly inside the 3D cell volume. We neglected possibility of more complex conditions for the network and cytosol flow at the plasma membrane that could be something between slip and stick conditions. These additional assumptions could be relaxed without changing main results in the thin-cell limit, for example, we could in principle use Navier slip law at the ventral boundary instead of the uniform adhesion drag in the 3D volume, but we wish to avoid more complex derivations.

The model (Eqs S1-5) can be scaled and non-dimensionalized as follows. We normalize$\Phi$by 1; for consistency we use the notation.$M=\bar{M}m$, where$\bar{M}$is the average myosin concentration. We use the thin cell approximation justified below (for the case of a high membrane tension): the average height of the cell is much smaller than the length and width of the lamellipodium,$H\sim\varepsilon L$, where$L$is the characteristic length/width, and$\varepsilon<<1$. We rescale the spatial coordinates as follows:$X=Lx,Y=Ly,Z=\varepsilon Lz,H=\varepsilon Lh$. The scale of velocity can be estimated from balancing the viscous and myosin contraction terms in (Eq S2):$U=\sigma\bar{M}L/\eta$. Thus,$U_{i}=Uu_{i},V_{i}=Uv_{i},W_{i}=\varepsilon Uw_{i},i=n,s$.Time can be rescaled as:$T=\left(L/U\right)t=\left(\eta/\sigma\bar{M}\right)t$. The scale of pressure can be estimated from balancing the pressure and Darcy terms in (Eq S3):$\bar{P}=KLU=\sigma K\bar{M}L^{2}/\eta$, and$P=\bar{P}p$.

When the cell height changes slowly in the x-y plane ($\partial h/\partial x,\partial h/\partial y<<1$), the z-components of the network and cytosol flow velocities can be neglected in the zeroth order approximation with respect to$\varepsilon$:$w_{n}=w_{s}=0$. After introducing notations$\nabla_{\perp}=\left(\partial/\partial x,\partial/\partial y,0\right)$,$\mathbf{u}_{i}=\left(u_{i},v_{i},0\right)$,$i=n,s$equations (Eqs S1-5) in the zeroth order approximation with respect to$\varepsilon$take the following form:

, (Eq S1’)

, (Eq S2’)

, (Eq S3’)

$\frac{\partial m}{\partial t}=\left(\frac{D\eta}{\sigma\bar{M}L^{2}}\right)\nabla_{\perp}^{2}m-\nabla_{\perp}\cdot\left(\mathbf{u}_{n}m\right)$, (Eq S4’)

$\nabla_{\perp}^{2}h=\left(\frac{\sigma K\bar{M}L^{3}}{\eta\varepsilon\gamma}\right)p$at$z=h\left(x,y,t\right)$. (Eq S5’)

Importantly, the system of equation becomes 2D in the thin-cell approximation, justifying the widely use 2D approximation for flat dynamic lamellipodia. As equation (Eq S5’) demonstrates, this approximation is valid in the limit of the high membrane tension, when non-dimensional combination of parameters$\left(\sigma K\bar{M}L^{3}/\eta\varepsilon\gamma\right)$is on the order of unity or smaller,$\gamma\sim\left(\sigma K\bar{M}L^{3}/\eta\varepsilon\right)$. Mathematically, this means that the membrane tension has to be high enough so that parameter$\varepsilon\sim\left(\sigma K\bar{M}L^{3}/\eta\gamma\right)<<1$. Physically, this means that if the membrane tension is high enough, it flattens the dorsal cell surface against the hydrostatic pressure of the cytosol. In fact, more relaxed inequality leads to a 2D class of models (Oliver et al., 2005), but here we wish to avoid complex derivations.

The validity of the 2D approximation is further supported by the observations of lamellipodial fragments that do not have a cell body. Their motility properties are very similar to those of the whole cell (Ofer et al., 2011). The fragments are extremely flat; even in their rear part, which is thicker than the front, they are only about one micron-thick, an order of magnitude less than their length and width (Ofer et al., 2011). There are many suggestions that the lamellipodium is the autonomous mechanical engine of the cell, while the 3D cell body simply rides passively atop of the lamellipodial actomyosin mesh (Rafelski and Theriot, 2004), but even taking the cell body into account does not change the thin-cell approximation, as the cell body thickness is but a few microns. Thus, the essential mechanics of the cell crawling on flat 2D surfaces is described well by the 2D model.

Furthermore, Rubinstein et al. (2009) estimated that Darcy friction between the cytosol and porous cytoskeletal network is roughly an order of magnitude smaller than effective viscous deformation forces within the cytoskeletal network:

$KL^{2}/\eta<<1$. This strong inequality leads to the significant simplification of the model. Indeed, as small termsand$\left(\frac{KL^{2}}{\eta}\right)\left(\mathbf{u}_{n}-\mathbf{u}_{s}\right)$can be neglected in equation (Eq S2’) compared to the other terms of the order of unity in this equation (pressure and Darcy friction are weaker than the effective viscous deformation, adhesion and myosin contraction forces), the equations for the network mechanics can be uncoupled from the equations for the cytosol: approximate equations

$\nabla_{\perp}^{2}\mathbf{u}_{n}+\nabla_{\perp}m=\left(\frac{\xi L^{2}}{\eta}\right)\mathbf{u}_{n}$, (Eq S1’’)

$\frac{\partial m}{\partial t}=\left(\frac{D\eta}{\sigma\bar{M}L^{2}}\right)\nabla_{\perp}^{2}m-\nabla_{\perp}\cdot\left(\mathbf{u}_{n}m\right)$, (Eq S2’’)

can be solved independently from the equations for the cytosol:

, (Eq S3’’)

, (Eq S4’’)

$\nabla_{\perp}^{2}h=\left(\frac{\sigma K\bar{M}L^{3}}{\eta\varepsilon\gamma}\right)p$at$z=h\left(x,y,t\right)$. (Eq S5’’)

Note that if one wants information about the volume fraction, pressure and cytosol flow, one needs to solve Eqs (S3’’-5’’) together with Eqs (S1’’-2’’). However, Eqs (S1’’-2’’) are self-consistent: there is no need to know pressure or actin volume fraction to find the myosin and network flow distributions. Eqs (S1’’-2’’) constitute the 2D model in the main text that we use. This argument supports the idea that there exists the experimentally relevant limiting case, in which the full 3D model is reduced to the 2D model, in which the pressure can be neglected.

Lastly, let us note that we did not discuss the very difficult problem of the boundary condition at the contact line – intersection of the dorsal and ventral surfaces. Respective physics is not well understood, and majority of existent 2D models use the free boundary conditions similar to that used in our model here (Oliver et al., 2005).

***Adding actin dynamics to the model.***

There could be, of course, a pressure term in an equation for a compressible medium as well. In order to do consider effects of such term, as well as consequences of possible density dependence of the actin viscosity, we explicitly introduced dynamic actin densities into the ZS model. Dynamics of actin density are governed by a combination of the F-actin disassembly and drift with the same velocity, as that in the equation for myosin. To consider the actin dynamics quantitatively, we added the following equation for the actin density, *F*, to the model equations for the ZS model described in the main text:

$\partial_{T}F=-\nabla\cdot\left(\text{U}F\right)-k_{dis}F$ (Eq S6)

on $\Omega\left(t\right)$. The boundary condition we used was:$F|_{\partial\Omega\left(t\right)}=F_{0}$. The equation for the velocity (force-balance equation) changed as follows:

$\eta\nabla\cdot\left(\left(1+\frac{\varepsilon F}{F_{0}}\right)\nabla\text{U}\right)+\sigma\nabla\cdot\left(M\hat{I}\right)-\zeta\nabla\cdot\left(F\hat{I}\right)-\xi\text{U}=0$ (Eq S7)

With respective change in the boundary condition:

$\text{n}\cdot\left(\eta\left(1+\frac{\varepsilon F}{F_{0}}\right)\nabla\text{U}+\sigma M\hat{I}-\zeta F\hat{I}\right)|_{\partial\Omega\left(t\right)}=0$ (Eq S8)

To investigate implications of such actin dynamics, we accounted for the dependence of actin viscosity on actin density (reflected in the factor$\left(1+\frac{\varepsilon F}{F_{0}}\right)$, assumed to be proportional to the actin density) and the effective swelling pressure (reflected in the term$\zeta F\hat{I}$, also assumed to be proportional to the actin density). We found that the model worked well with simple protrusion rate of the form:$V_{p}=V_{0}\frac{A_{0}}{\left|\Omega\right|}-K\left|\Omega\right|$. We simulated the model using the non-dimensional parameter values$v_{0}=1,\mu_{tot}=1.5708,\alpha=0.5$, for which the ZS model without actin predicted the cell moving straight. We found that as with our original model, the expanded model with explicit actin densities has stationary, translating, and rotating steady states. Namely, for small values of the swelling pressure ($\varepsilon=0.01,k_{dis}=1,\zeta=0.5$), the simulated cell moved straight and steadily. For greater values of the swelling pressure ($\varepsilon=0.01,k_{dis}=1,\zeta=1$), the simulated cell remained stationary and symmetric. This is easy to understand because the swelling pressure effectively counteracts the myosin contractile stress. If, on the other hand, we increase the protrusion rate (in the simulations we did that by changing the protrusion rate to$V_{p}=V_{0}\frac{A_{0}}{\left|\Omega\right|}-K\left(\left|\Omega\right|-A_{0}\right)$, or effectively adding the term$KA_{0}$to the protrusion rate), we expect the rotating cell, which indeed was the case ($\varepsilon=0.01,k_{dis}=1,\zeta=0.14$).

***Note on the anti-crowding terms in the model equations.***

It is possible that the anti-crowding effects come from an attenuation of the myosin stress when the myosin density is too high. To explore this possibility, the factor (1-M/M_tot_) was removed from the advection term of the equation for myosin and inserted into the active stress term of the equation for velocity: $\nabla\cdot\mathbf{(}M\left( 1-\frac{M}{M_{\max}} \right)\mathbf{I})$, with the same $M_{\max}$ that was previously used in the advection term (the ‘anti-crowding’ term for diffusion was either kept as before, or removed in that case, with no significant effect on the results). Simulations were run for three cases depicted in Fig. 1, and the results were compared against the original results for several metrics as summarized in three tables below:

**ZS: Stationary cell** $\left( \mathbf{v}_{\mathbf{0}}\mathbf{,}\boldsymbol{\mu}_{\mathbf{tot}}\boldsymbol{, \alpha} \right)\boldsymbol{=(2.5 , 0.125\pi, 0.5)}$

| Measured parameter | Nonlinear advection $\nabla\cdot\mathbf{(v}\left( 1-\frac{M}{M_{\max}} \right)M)$ | Nonlinear contractility $\nabla\cdot(M\left( 1-\frac{M}{M_{\max}} \right)\mathbf{I})$ |
| --- | --- | --- |
| Aspect ratio | 1.00 | 1.00 |
| Steady-state area of the cell | 3.49 | 3.50 |
| max(Myosin) at steady-state | 0.13 | 0.13 |
| min(Myosin) at steady-state | 0.09 | 0.09 |

**ZV: Translating cell** $\left( \mathbf{v}_{\mathbf{0}}\mathbf{,}\boldsymbol{\mu}_{\mathbf{tot}}\boldsymbol{, \alpha} \right)\boldsymbol{=(2.5 , 2\pi, 0.5)}$

| Measured parameter | Nonlinear advection $\nabla\cdot\mathbf{(v}\left( 1-\frac{M}{M_{\max}} \right)M)$ | Nonlinear contractility $\nabla\cdot(M\left( 1-\frac{M}{M_{\max}} \right)\mathbf{I})$ |
| --- | --- | --- |
| Translational cell speed | 0.19 | 0.18 |
| Aspect ratio | 1.63 | 1.33 |
| Steady-state area of the cell | 3.85 | 3.84 |
| max(Myosin) at steady-state | 13.96 | 11.35 |
| min(Myosin) at steady-state | 0.01 | 0.05 |

**ZS: Rotating cell** $\left( \mathbf{v}_{\mathbf{0}}\mathbf{,}\boldsymbol{\mu}_{\mathbf{tot}}\boldsymbol{, \alpha} \right)\boldsymbol{=(2.5 , 0.75\pi, 0.5)}$

| Measured parameter | Nonlinear advection $\nabla\cdot\mathbf{(v}\left( 1-\frac{M}{M_{\max}} \right)M)$ | Nonlinear contractility $\nabla\cdot(M\left( 1-\frac{M}{M_{\max}} \right)\mathbf{I})$ |
| --- | --- | --- |
| angular velocity | 0.58 | 0.37 |
| Radius of rotation | 1.12 | 1.11 |
| Aspect ratio | 2.13 | 1.71 |
| Steady-state area of the cell | 2.74 | 2.86 |
| max(Myosin) at steady-state | 9.22 | 5.35 |
| min(Myosin) at steady-state | 0.01 | 0.03 |

The results indicated that the stationary, rotational, and unidirectional behaviors are qualitatively the same as in our original models, and even quantitative characteristics of the solutions are close to the original. Of course, the boundaries between the corresponding regions in the state diagrams change, but only a little.

***Derivations and assumptions related to the protrusion rates.***

We originally used different expressions for the boundary protrusion rate V_p_ in the two models for the following reason. The dependence of V_p_ on myosin is essential in the ZV model, as has been discussed in detail in the main text: without it, the cell never moves and changes shape in this version of the model. In the ZS model, the cell moves and changes shape with V_p_ independent of myosin, and so we decided to test this simplest case. The usage of different exponents (n=0 in ZV and n=2 in ZS) was due to the fact that we initially wanted to find the smallest integer exponent n for which the model predicted a stable behavior. It turned out that n=0 was the smallest for the ZV model, and n=2 for the ZS model. By using a myosin-dependent V_p_ in the ZS model and increasing n to n=2 in the ZV model, one can unify the models, simplify the number of choices, and also demonstrate robustness of the models. So we explored the models with the same protrusion velocity,

$V_{p}=V_{0}\left( \frac{A_{0}}{A} \right)\left( 1+\frac{M|_{\partial\Omega(t)}}{M_{0}} \right)^{-1}-K(A-A_{0}\left( \frac{A_{0}}{A} \right)^{2})$. (Eq S9)

We found that this resulted in an essentially same state diagram for the ZV model:

The state diagram of the ZS model also remains qualitatively similar to that in the main text, with the following differences: i) the border between the stationary and moving states is now slightly dependent on M, like in our ZV diagram; ii) the region of rotating states has shrunk somewhat; 3) cells move faster due to the enhanced coupling between front velocities and myosin.

Biophysics underlying the expression for the protrusion rate$V_{p}$is based on the force balance at the lamellipodial boundary. As the lamellipodium is essentially flat, the force balance at this boundary is quite simple: There is an outward polymerization force exerted by growing actin filament tips abutting the plasma membrane,$F_{pol}$, complemented by the effective elastic resistance of the actin cortex and the cell body,$F_{res}$, balanced by the inward membrane tension,$T$ (all forces are per micron of the cell edge.) Thus,

$F_{pol}+F_{res}=T$ (Eq S10)

It was shown that membrane bending forces are negligible compared to the effect of the membrane tension (Kozlov and Mogilner, 2007). We also assume that adhesion between the actin network and plasma membrane can be neglected.

The polymerization force is proportional to the density of growing actin filaments per unit length of the cell edge,$N$. As was noted in our supplemental material, we assume that the actin tip density at the boundary depends on the cell area (Lieber et al., 2013) because of a limited amount of actin regulatory proteins; specifically, we assume that this density is inversely proportional to the lamellipodial area:$N=N_{0}/A$, where$A$is the lamellipodial area and$N_{0}$is the proportionality coefficient. Furthermore, the polymerization force is a decreasing function of the protrusion rate. In the limited range of the protrusion rates, this dependency can be approximated with the linear function:

$F_{pol}=NF_{p}-\vartheta V_{p},N=\frac{N_{0}}{A}$ (Eq S11)

where$\vartheta$is the proportionality coefficient and$F_{p}$is the stall force per filament.

The membrane tension is spatially uniform, and it is an increasing function of the lamellipodial area (Lieber et al., 2013). Mechanically, this is likely due to the fact that the increase of the lamellipodial area, while the total plasma membrane area remains constant on the relevant time scale, makes the membrane flatter in the cell body region, thus squeezing the viscoelastic cell body and creating the mechanical tension. In our model, we assume that the membrane tension is proportional to the lamellipodial area:$T\sim A$. The mechanical effect from the elastic resistance of actin cortex and the cell body that counters too much shrinking of the cell area has to increase sharply with the decrease of the lamellipodial area. We assume that respective resistance force is inversely proportional to the *n*^th^ degree of the lamellipodial area:$F_{res}\sim A^{-n}$.We define the characteristic cell area$A_{0}$as the area at which the opposing membrane tension and elastic cell body resistance forces are balanced:$T=F_{0}\left(A/A_{0}\right)=F_{0}\left(A_{0}/A\right)^{n}=F_{res}$. Here$F_{0}$is this balanced force magnitude at the characteristic cell area. Thus:

$T=F_{0}\left(A/A_{0}\right)$ (Eq S12)

$F_{res}=F_{0}\left(A_{0}/A\right)^{n}$ (Eq S13)

By substituting Eqs (S11-13) into Eq (S10), we arrive at the expression for the protrusion rate in the ZS model:

$V_{\text{p}}=V_{0}(A_{0}/A)-K(A-A_{0}(A_{0}/A)^{n})$ (Eq S14)

where$V_{0}=\frac{N_{0}F_{p}}{\vartheta A_{0}},K=\frac{F_{0}}{\vartheta A_{0}}$.

As we wrote above, the protrusion rate has to be an increasing function of the local actin density. In some special cases, the actin density and the protrusion rate are, in fact, almost uniform around the cell boundary: this was the reported case for keratocytes moving on weakly adhesive surfaces (Barnhart et al., 2011). Thus, we made the respective simplest assumption for the ZS model.

The expression for the protrusion rate in the ZV model is based on a similar force balance. In the ZV model, the nontrivial solutions require a non-uniform rate of protrusion. In majority of cases, indeed, the rate of protrusion is maximal at the leading edge and decreases to the sides and rear of the cell (Barnhart et al., 2011). Coincidentally, myosin density increases towards the rear of the motile cell. A recent study (Lomakin et al., 2015) strongly suggests that myosin contraction locally destroys the protrusive branched actin network, and so the branched actin density and protrusion rate are decreasing function of the local myosin density. This is reflected by the term V_p_A_0_/(A(1+M/M_0_)).

***Notes on the myosin transport.***

The assumption behind the myosin transport equation in our model is that myosin binding kinetics are fast compared to the transport terms and, are therefore, rapidly equilibrated. In that case, two equations for two sub-populations of myosin, one – myosin bound to actin network and drifting with the network, another – diffusing in the cytosol, have the form:

$\begin{matrix} \partial_{t}m_{b}=-\nabla\cdot(\mathbf{u}m_{b})+k_{on}m_{f}-k_{off}m_{b} \\ \partial_{t}m_{f}=\nabla\cdot(d\nabla m_{f})-k_{on}m_{f}+k_{off}m_{b} \end{matrix}$ (Eq S15)

When the kinetics is fast,$k_{on}m_{f}\approx k_{off}m_{b}$, and this system of equations reduces to the single equation for the total local myosin,$m=m_{f}+m_{b}$:

,. (Eq S16)

To make sure that the approximation is valid, we simulated the models with myosin transport equation (2) in the main text replaced by the system of equations (Eq S15), with $k_{on},k_{off} >>1$. Specifically, we simulated the model with myosin transport described by Eq S15, in three cases:

| Model | Parameter set $\left( v_{0} , \mu_{tot} , \alpha\right)$ | Mechanical state |
| --- | --- | --- |
| ZS | $\left( 7.5 , \pi/4 , 1 \right)$ | Stationary |
| ZS | $\left( 7.5 , \pi/2 , 1 \right)$ | Rotating |
| ZV | $\left( 7.5 , \pi/2 , 1 \right)$ | Translating |

Results indicated that the model formulated in terms of two myosin sub-populations is essentially identical to the corresponding model described in the main text when $k_{on},k_{off} >>1$.

Our models have common features with the one in (Wolgemuth et al., 2011). In the latter, steadily migrating solutions were not found. Out of 3 types of solutions found in (Wolgemuth et al., 2011), one – a stationary cell at low contractility – is the same as in our case. Another – when contractility is higher – created a cell ‘pinched’ into the dumbbell shape. We observed such shapes transiently, but they rapidly became rotating. The third solution corresponded to parameter α being very small, in which case of cell developed into a 'sausage’-like shape. Our model with small α (~ 0.1) still exhibits steadily motile (straight and rotating) shapes.

We believe that these discrepancies are caused by the differences between our model and that in (Wolgemuth et al., 2011). One significant difference is that in the transport equation system of (Wolgemuth et al., 2011), Eq S15, the free myosin instantaneously levels off, implying the infinite diffusion coefficient. The fast diffusion assumption means that the time of dissipation of gradients of free myosin is much shorter than the time scale of the model defined as L/V_0_, i.e. L^2^/D << L/V_0_, or V_0_L/D << 1. In our models, V_0_L/D=v_0_ is the dimensionless version of V_0_. Thus, if it were not for other differences with our models, the model of (Wolgemuth et al., 2011) would be equivalent to the limit of slow protrusion in our models. In this limit, the ZV model has only stationary solutions, even for asymmetric myosin, and the ZS model does not have rotational solutions for α = 1. There is another difference caused by the finiteness of diffusion in our models, which likely explains why the unidirectional (and rotational, for α = 0.5) motility in the ZS model for small v_0_ was not observed in the model of (Wolgemuth et al., 2011). Because the diffusion coefficient is finite in our models, we account for volume excluded by myosin to avoid the singularities, which apparently do not occur if the diffusion is infinitely fast. As a result, the bound myosin does not form tight, delta-function-like aggregates for intermediate values of myosin, making the cell boundary more dynamic. Finally, there are minor differences in the expressions for the boundary protrusion velocity used in our study and (Wolgemuth et al., 2011).

Barnhart EL, Lee KC, Keren K, Mogilner A, Theriot JA. An adhesion-dependent switch between mechanisms that determine motile cell shape. PLoS Biol. 2011 9:e1001059.

Cogan NG, Guy RD. 2010. Multiphase flow models of biogels from crawling cells to bacterial biofilms. HFSP J. 4(1):11—25

Dembo M, Harlow F. 1986. Cell motion, contractile networks, and the physics of interpenetrating reactive flow. Biophys. J. 50(1):109--21

Drew DA, Segel LA. 1971. Averaged equations for two‐phase flows. Stud. Appl. Math. 50(3):205--31

Kozlov MM, Mogilner A. Model of polarization and bistability of cell fragments. Biophys J. 2007 Dec 1;93(11):3811-9.

Kuusela E, Alt W. 2009. Continuum model of cell adhesion and migration. J. Math. Biol. 58(1--2):135--61

Lieber AD, Yehudai-Resheff S, Barnhart EL, Theriot JA, Keren K. Membrane tension in rapidly moving cells is determined by cytoskeletal forces. Curr Biol. 2013 23:1409-17.

Lomakin AJ, Lee KC, Han SJ, Bui DA, Davidson M, Mogilner A, Danuser G. Competition for actin between two distinct F-actin networks defines a bistable switch for cell polarization. Nat Cell Biol. 2015 17:1435-45.

Ofer N, Mogilner A, Keren K. Actin disassembly clock determines shape and speed of lamellipodial fragments. Proc Natl Acad Sci U S A. 2011 108:20394-9.

Oliver JM, King JR, McKinlay KJ, Brown PD, Grant DM, et al. 2005. Thin-film theories for two-phase reactive flow models of active cell motion. Math. Med. Biol. 22(1):53—59

Rafelski SM, Theriot JA. Crawling toward a unified model of cell mobility: spatial and temporal regulation of actin dynamics. Annu Rev Biochem. 2004 73:209-39.

Rubinstein B, Fournier MF, Jacobson K, Verkhovsky AB, Mogilner A. Actin-myosin viscoelastic flow in the keratocyte lamellipod. Biophys J. 2009 97:1853-63.

Wolgemuth CW, Stajic J, Mogilner A. Redundant mechanisms for stable cell locomotion revealed by minimal models. Biophys J. 2011 101:545-53.
